# When college students perceive GAI feedback as credible: the role of perceived GAI feedback credibility in promoting deep learning through cognitive engagement

**DOI:** 10.3389/fpsyg.2026.1986129

**Published:** 2026-10-22

**Authors:** Wen Guo, Xiaojian Ren

**Affiliations:** Shandong Women’s University, Jinan, China

**Keywords:** cognitive engagement, college students’ psychological processes, deep learning, educational psychology, GAI feedback credibility

## Abstract

Generative artificial intelligence (GAI) has rapidly developed in higher education and has raised new questions from an educational psychology perspective. Why does GAI feedback promote meaningful learning for some college students but fail to produce similar learning benefits for others? To answer this question, this study develops a theoretical framework based on Information Processing Theory, Elaboration Likelihood Model, and Constructivist Learning Theory. This study examines how perceived GAI feedback credibility influences college students’ deep learning and explores the mediating role of cognitive engagement in this relationship. This study adopts a stratified simple sampling method and collects questionnaire data from 506 undergraduate students and uses structural equation modeling (SEM) to examine the proposed model. The results show that perceived GAI feedback credibility has positive relationships with both cognitive engagement and deep learning. In addition, cognitive engagement plays a significant mediating role between perceived GAI feedback credibility and deep learning, suggesting that credible GAI feedback may support deep learning by encouraging college students to engage in cognitive processing and reflective thinking. This study extends research on learning psychology in GAI-supported learning environments by explaining how college students evaluate and process GAI feedback. The findings indicate that the influence of GAI feedback on learning does not simply result from the technology itself. Instead, college students’ judgments of feedback credibility and their following cognitive engagement play important roles in shaping deep learning processes.

## 1 Introduction

Generative artificial intelligence (GAI) has rapidly developed in higher education and has introduced a new feedback context in which college students increasingly interact with GAI, such as ChatGPT, DeepSeek, and Doubao, during learning activities. From an educational psychology perspective, feedback is not simply external information provided to learners. Instead, the influence of feedback depends on how college students perceive, understand, and cognitively process information. Traditionally, feedback has mainly occurred through teacher-student interactions and has supported learning by helping learners identify knowledge gaps, regulate cognitive processes, and develop deeper understanding. However, with the emergence of large language model-based systems such as ChatGPT and DeepSeek, college students increasingly need to evaluate feedback provided by non-human information sources (Huang et al., 2026). Although GAI systems can generate immediate explanations, suggestions, and evaluations based on college students’ questions and learning contexts (Albadarin et al., 2024), the psychological value of GAI feedback does not automatically come from the availability of technology. Instead, it depends on college students’ judgments about the credibility of GAI feedback information and their willingness to engage in cognitive processing (Henderson et al., 2025).

Based on this perspective, this study introduces the concept of perceived GAI feedback credibility, which refers to college students’ subjective evaluations of the reliability, accuracy, and dependability of GAI feedback during learning activities. It is important to clarify that perceived GAI feedback credibility does not represent the objective correctness of GAI feedback information. Instead, it reflects how college students understand and evaluate GAI feedback as a source of learning information. From the perspective of cognitive psychology, college students are not passive recipients of external information. Rather, they actively evaluate, understand, and integrate information based on their previous experiences, knowledge structures, and learning contexts (Escalante et al., 2023). Such evaluations influence whether college students are willing to invest cognitive resources to further examine, process, and use the provided GAI feedback. Therefore, examining perceived GAI feedback credibility does not aim to evaluate the effectiveness of GAI technology itself (Robinson, 2026). Instead, it aims to understand how college students make information judgments and regulate cognitive processing when they interact with new types of feedback sources (Johnston et al., 2024).

Although college students’ perceptions of GAI feedback credibility may influence their learning processes, an important question remains, how does the psychological evaluation of GAI feedback relate to college students’ cognitive activities during learning? Perceiving an information source as credible does not automatically lead to meaningful learning. Educational psychology research has consistently emphasized that learning depends on how college students actively attend to, process, and integrate information into their existing knowledge structures. Even when college students consider GAI feedback reliable, they may accept the provided information without further reflection, evaluation, or connection with their existing understanding (Zapata et al., 2025). In this situation, the potential value of GAI feedback may not be fully realized because meaningful learning requires college students to actively participate in cognitive activities. Therefore, the role of perceived GAI feedback credibility does not only concern whether college students accept GAI feedback information. More importantly, it concerns whether this psychological evaluation can encourage college students to engage in deeper cognitive processing of GAI feedback information.

Accordingly, cognitive engagement can serve as an important psychological mechanism for explaining how GAI feedback influences learning. Cognitive engagement refers to the extent to which college students actively invest mental effort and cognitive resources to process, evaluate, and understand information during learning. Unlike simple learning participation or behavioral engagement (Fredricks et al., 2004), cognitive engagement focuses on how college students think about and process information (Kasneci et al., 2023). In GAI-supported learning environments, when college students perceive GAI feedback as credible, they may become more willing to actively examine and process such feedback rather than simply consider it as the final answer. This psychological state encourages college students to evaluate the relevance and accuracy of GAI suggestions, compare them with their own understanding, and actively participate in the feedback processing process. Therefore, perceived GAI feedback credibility may promote college students’ active cognitive processing by increasing cognitive engagement (Abbas et al., 2024). Furthermore, cognitive engagement not only reflects how college students process and understand GAI feedback but also represents an important process through which information processing contributes to deep learning.

In traditional learning environments, deep learning is influenced by instructional design, teacher guidance, and college students’ learning strategies (Marton and Säljö, 1976). However, the involvement of GAI provides new possibilities for supporting learning development. GAI can provide immediate cognitive support, but whether such support can promote deep learning depends on whether college students actively process and evaluate GAI feedback rather than simply accept GAI outputs. If college students consider GAI as a substitute for their own thinking, GAI may lead to passive dependence and surface learning. In contrast, if college students use GAI as a tool to support their own thinking and learning activities, GAI feedback may promote deeper knowledge understanding and application (Chan and Hu, 2023). Therefore, understanding how perceived GAI feedback credibility influences deep learning through cognitive engagement is important for explaining learning processes in the era of GAI.

Although research on the educational application of GAI has increased rapidly, existing studies still have several limitations. First, many studies (Yusuf et al., 2024) focus on whether college students use GAI, whether college students accept GAI, or how to increase GAI adoption intention. However, fewer studies examine how college students psychologically evaluate GAI information. Second, previous studies have mainly focused on the relationship between GAI use and learning outcomes, including academic achievement, higher-order thinking, and learning performance, while relatively less attention has been given to the psychological and cognitive processes through which GAI influences learning (Chen and Cheung, 2025; Han et al., 2025). Although recent research has begun to examine learner-related factors and human-GAI interaction, how college students evaluate and cognitively process GAI feedback remains insufficiently understood (Bai and Wang, 2025; Boolzen et al., 2026). Third, current research has not fully explained how perceived GAI feedback credibility influences college students’ active cognitive processing. Existing studies have shown that GAI can provide personalized support, immediate feedback, and cognitive assistance, which may facilitate students’ engagement and learning outcomes (Kasneci et al., 2023; Han et al., 2025). However, research on cognitive offloading suggests that reliance on external cognitive resources may also reduce individuals’ internal cognitive effort and alter information-processing strategies (Risko and Gilbert, 2016). Therefore, it remains unclear whether perceiving GAI feedback as credible encourages deeper cognitive engagement or leads to reduced cognitive effort through reliance on GAI-generated information. Finally, recent studies further indicate that GAI-supported learning involves complex interactions among technological functions, college students’ cognition, and knowledge construction. Existing studies have examined the effects of GAI on learning outcomes (Cong-Lem and Fan, 2026), the design of GAI-supported learning environments based on constructivist principles (Liu et al., 2026), the importance of metacognitive abilities when college students interact with GAI systems (Levin et al., 2025), and how human-GAI interaction influences learning processes (Liang et al., 2026). These studies suggest that the educational value of GAI depends not only on the information provided by GAI systems but also on how college students evaluate, process, and actively engage with GAI information.

Based on self-reported questionnaire data from college students, this study develops a theoretical model including perceived GAI feedback credibility, cognitive engagement, and deep learning to examine how college students’ evaluations of GAI feedback relate to their cognitive processing and learning experiences in GAI-supported learning environments. By examining the psychological pathway from perceived GAI feedback credibility to deep learning through cognitive engagement, this study provides further insight into how college students interpret GAI feedback and regulate their cognitive activities during learning. The findings provide some implications for explaining the psychological processes involved in college students’ interactions with GAI feedback.

## 2 Literature review

### 2.1 Theoretical foundation and the proposed theoretical framework

This study integrates the Elaboration Likelihood Model, Information Processing Theory, and Constructivist Learning Theory as complementary theoretical perspectives to explain how perceived GAI feedback credibility relates to cognitive engagement and deep learning in GAI-supported learning environments. These theories share the assumption that learners actively participate in the learning process rather than simply receive external information. However, each theory emphasizes different aspects of this process. The Elaboration Likelihood Model explains how learners evaluate information sources and decide whether to engage in deeper information processing. Information Processing Theory explains how learners attend to, organize, and integrate external information through cognitive activities. Constructivist Learning Theory further explains how learners construct understanding by connecting new information with existing knowledge. Therefore, integrating these perspectives provides a more comprehensive explanation of how college students interact with GAI feedback than relying on any single theoretical perspective.

The Elaboration Likelihood Model proposed by Petty and Cacioppo (1986) provides an important explanation for how college students’ evaluations of GAI feedback credibility may influence their willingness to engage with feedback information. The theory suggests that individuals process information through different levels of elaboration depending on their motivation, cognitive ability, and evaluation of the information source. When individuals perceive information as valuable and reliable, they are more likely to carefully examine and process the information through the central route. In contrast, when individuals have limited motivation or perceive information as less valuable, they may rely more on simple cues and invest fewer cognitive resources in information processing. In GAI-supported learning environments, GAI feedback represents a new type of information source that differs from traditional human feedback. Although GAI feedback can provide immediate explanations, suggestions, and evaluations, college students still need to judge whether such feedback is reliable and useful before further processing it. Therefore, the Elaboration Likelihood Model helps explain why perceived GAI feedback credibility may influence college students’ willingness to allocate cognitive resources and engage with GAI feedback.

However, the Elaboration Likelihood Model alone cannot fully explain how college students process GAI feedback after they evaluate its credibility. To explain this cognitive process, Information Processing Theory provides an additional perspective. Information Processing Theory explains how individuals receive, encode, store, and use external information. Atkinson and Shiffrin (1968) proposed the multi store model of human memory, suggesting that information processing involves sensory memory, short term memory, and long term memory. From this perspective, external information cannot directly become meaningful knowledge. Instead, learners need to actively select, organize, and integrate information through cognitive activities. Mayer (2008) further emphasized that meaningful learning depends on learners’ active cognitive processing rather than simple exposure to information. In the context of GAI-supported learning, GAI feedback functions as an external information input. Nevertheless, such feedback cannot directly produce learning experiences. College students need to evaluate, interpret, compare, and organize the information through their own cognitive activities. Therefore, Information Processing Theory helps explain why college students who perceive GAI feedback as credible may show greater cognitive engagement, as they may be more willing to invest mental effort in processing and understanding the provided feedback.

At the same time, Information Processing Theory mainly focuses on how individuals process information, but it does not fully explain how processed information develops into deeper understanding and knowledge construction. Constructivist Learning Theory provides an additional explanation for this aspect. Based on Piaget’s cognitive constructivism and Vygotsky’s sociocultural theory, Constructivist Learning Theory emphasizes that learners actively construct knowledge through interaction with information, experiences, and external support. Piaget (1952) argued that learners develop knowledge by adjusting their existing cognitive structures through assimilation and accommodation. Vygotsky and Cole (1978) further emphasized that cognitive development occurs through social interaction and the use of cultural tools. From this perspective, GAI feedback serves as a learning resource that can support knowledge construction, but meaningful learning depends on whether college students actively interpret and integrate the information into their existing knowledge structures. Therefore, Constructivist Learning Theory explains why cognitive engagement with GAI feedback may contribute to deeper learning experiences characterized by meaningful understanding, knowledge connection, and knowledge application.

Together, these three theoretical perspectives provide an integrated explanation of the learning mechanism associated with GAI feedback. The Elaboration Likelihood Model explains the initial evaluation process through which college students determine whether GAI feedback deserves further attention and processing. Information Processing Theory explains how college students cognitively process and organize GAI feedback after they decide to engage with it. Constructivist Learning Theory explains how such cognitive activities contribute to the construction of understanding and deeper learning experiences. Importantly, these perspectives do not represent separate theoretical stages. Instead, they describe interconnected aspects of the same learning process in which information evaluation, cognitive processing, and knowledge construction continuously influence one another. The three theoretical perspectives adopted in this study should not be understood as independent explanations for individual variables. Instead, they provide overlapping and complementary explanations for the psychological processes underlying learners’ interactions with GAI-generated feedback. Specifically, ELM explains not only how learners form credibility judgments about GAI feedback but also how such judgments influence their willingness to engage in further cognitive processing. Information Processing Theory provides a broader explanation of how learners attend to, elaborate on, and integrate GAI feedback information, thereby explaining both cognitive engagement and the development of deeper understanding. Constructivist Learning Theory further emphasizes learners’ active role in constructing meaning, which is closely related to both cognitive engagement and deep learning approaches. Together, these three theoretical perspectives offer an integrated explanation of how learners evaluate, process, and construct knowledge when interacting with GAI feedback. Rather than representing separate theoretical stages, they jointly explain the interconnected psychological processes through which perceived GAI feedback credibility is associated with cognitive engagement and deep learning.

Based on the above theoretical perspectives, this study further develops an *Information evaluation–Cognitive processing–Knowledge construction Framework* to explain the psychological processes through which college students interact with GAI feedback in GAI-supported learning environments (Figure 1). This framework suggests that GAI feedback does not directly lead to learning outcomes. Instead, it requires college students to actively participate in processes of information evaluation, cognitive processing, and knowledge construction. Specifically, when college students encounter GAI feedback, they first need to evaluate whether the information is credible and valuable, which influences whether they are willing to further attend to and process the feedback. Then, college students need to invest cognitive resources to understand, analyze, compare, and integrate the feedback information, allowing external information to connect with their existing knowledge structures. Finally, information that college students actively process can support deeper understanding and knowledge application.

**Figure 1:**
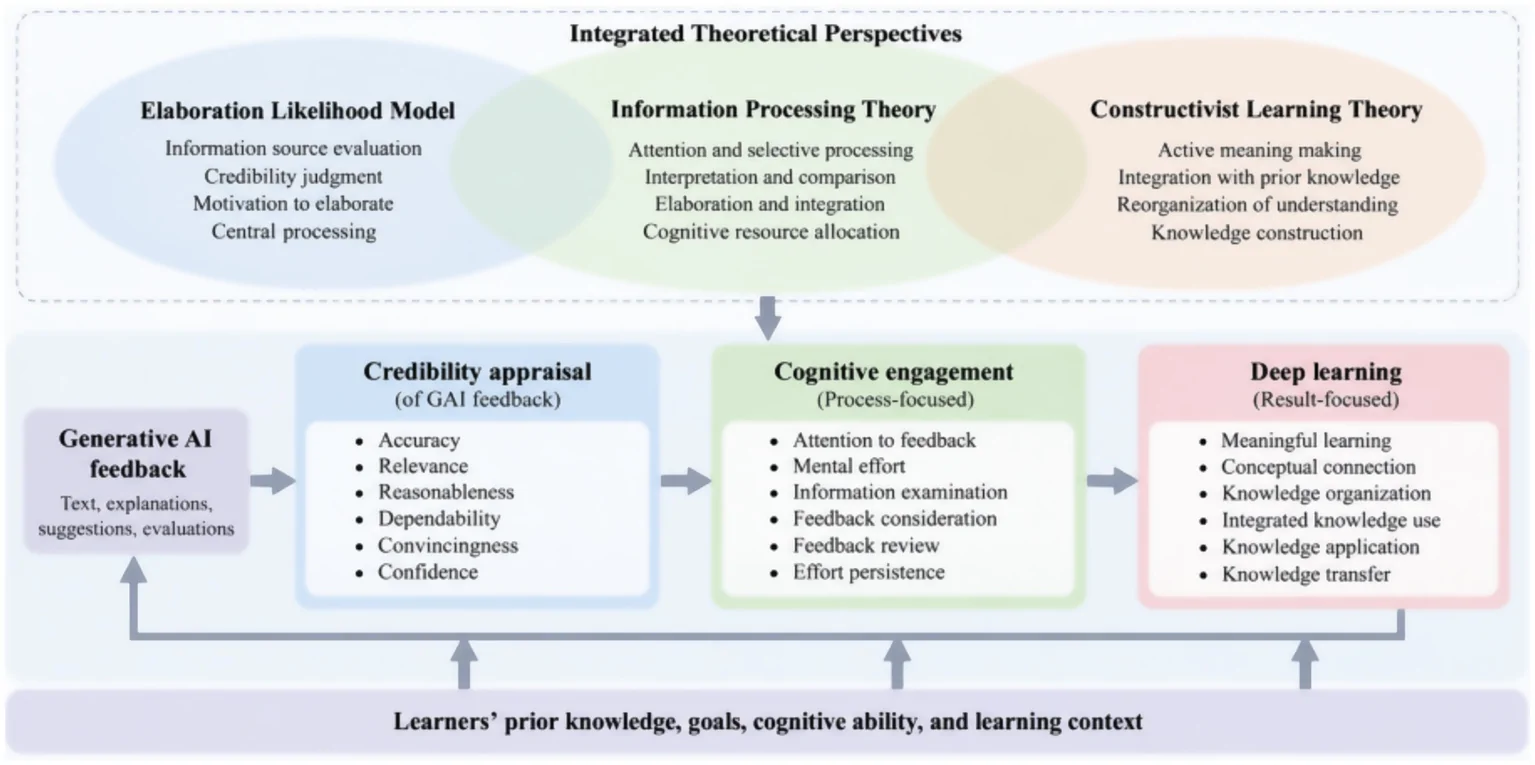
Theoretical framework of this study.

Following this psychological mechanism, this study selects perceived GAI feedback credibility, cognitive engagement, and deep learning as the core research constructs. First, the information evaluation process represents the starting point of college students’ interaction with GAI feedback. Therefore, it is necessary to examine how college students form credibility judgments about GAI feedback and how these judgments influence subsequent learning activities. Second, information evaluation itself does not automatically lead to learning development. College students still need to actively process the obtained information through cognitive activities. Therefore, cognitive engagement represents an important psychological process that connects information evaluation and learning experiences. Furthermore, the value of cognitive processing depends on whether college students can connect new information with existing knowledge and develop deeper understanding and application. Therefore, deep learning reflects an important learning experience associated with knowledge construction. Based on this theoretical logic, this study further examines how perceived GAI feedback credibility relates to deep learning and how cognitive engagement plays a role in this relationship.

### 2.2 Conceptual distinction among perceived GAI feedback credibility, cognitive engagement, and deep learning

Perceived GAI Feedback Credibility, Cognitive Engagement, and Deep Learning represent three distinct but related concepts in GAI-supported learning environments. Although they are examined within the same learning context, they reflect different psychological and learning dimensions. Specifically, Perceived GAI Feedback Credibility refers to students’ evaluations of the reliability, accuracy, and usefulness of GAI-generated feedback. Cognitive Engagement represents the cognitive process through which students actively attend to, process, analyze, and reflect on GAI feedback during learning. Deep Learning refers to the learning outcomes associated with deeper understanding, knowledge integration, and meaningful application of knowledge. In this study, these constructs were measured through students’ self-reported perceptions and experiences. Therefore, the measures capture students’ reported cognitive engagement processes and perceived deep learning outcomes rather than directly observing cognitive processes or objectively assessing learning performance.

It is important to distinguish between trust and perceived credibility in GAI-supported learning environments. Although these two concepts are closely related, they refer to different levels of psychological judgment. Generally, trust is a broader psychological concept that refers to whether learners are willing to believe in and rely on an GAI system and its recommendations (Pitts and Motamedi, 2025). For example, when students believe that “ChatGPT is a reliable learning tool and I am willing to use it to support my learning,” they demonstrate trust in the GAI system as a whole. Trust concerns the overall relationship between learners and GAI, including whether learners are willing to consider GAI as a dependable source of support during learning. In contrast, perceived credibility refers to learners’ evaluations of specific information provided by GAI (Pudasaini, 2026). It focuses on whether learners consider a particular piece of GAI feedback to be accurate, reliable, and valuable for learning. For example, when students receive GAI-generated feedback, they may ask: “Is this feedback accurate?” “Is this explanation consistent with my understanding?” or “Can this suggestion help me improve my learning?” These judgments are directed toward specific feedback rather than the GAI system as a whole. Therefore, the main difference between trust and perceived credibility is that trust concerns whether learners believe in and rely on the GAI system, whereas perceived credibility concerns whether learners consider specific GAI-generated information worthy of acceptance and further processing. During learning, students may perceive a specific piece of GAI feedback as credible without developing general trust in the GAI system. For example, students may consider ChatGPT’s explanation of a particular concept accurate, while still remaining cautious about using GAI to solve all types of learning problems. Based on this distinction, this study focuses on Perceived GAI Feedback Credibility rather than general trust in GAI. The reason is that this study does not examine whether students are willing to rely on GAI systems in general. Instead, it focuses on how students evaluate the reliability and value of GAI-generated feedback and how such evaluations are related to their subsequent cognitive processing and learning experiences. Therefore, perceived GAI feedback credibility more directly reflects the psychological process examined in this study, namely how learners evaluate GAI feedback information before engaging with it during learning.

Perceived GAI Feedback Credibility refers to students’ subjective judgment regarding whether GAI-generated feedback is reliable, accurate, and trustworthy (Appelman and Sundar, 2016). This construct focuses exclusively on learners’ perceptions of the feedback source and the information provided by GAI systems (Pudasaini, 2026). It captures whether students believe that GAI-generated feedback is credible enough to be considered and used during learning (Henderson et al., 2025). Importantly, perceived GAI feedback credibility does not indicate whether students actually engage with the feedback or whether their understanding of knowledge has changed. A student may perceive GAI feedback as highly credible but may not actively process the feedback or achieve deeper learning (Risko and Gilbert, 2016).

Cognitive Engagement refers to the mental effort and cognitive activities that learners invest when processing learning information (Fredricks et al., 2004). Unlike perceived GAI feedback credibility, which reflects learners’ evaluation of GAI-generated feedback, cognitive engagement reflects learners’ responses after receiving such feedback (Sinatra et al., 2015). It describes the process through which students actively examine, analyze, interpret, and evaluate information provided by GAI systems. Therefore, cognitive engagement focuses on how students process feedback, rather than whether the feedback is considered credible or what learning outcomes are eventually achieved.

Deep Learning refers to the quality of learning outcomes that learners achieve after engaging in learning activities (Reinhold et al., 2024). It reflects whether learners develop meaningful understanding, establish connections among concepts, integrate new knowledge into existing knowledge structures, and apply knowledge to new situations (Wang and Guo, 2025). Unlike cognitive engagement, which occurs during the process of information processing, deep learning reflects the results produced by that process. In other words, cognitive engagement describes the effort and activities involved in learning, whereas deep learning describes the depth and quality of understanding developed through learning (Yujie et al., 2026).

The distinction among these three constructs can therefore be summarized as follows: perceived GAI feedback credibility answers “How do students evaluate GAI feedback?”; cognitive engagement answers “How do students process and interact with GAI feedback information?”; and deep learning answers “What level of understanding and knowledge development do students achieve after learning?” Accordingly, this study proposes that students’ perceptions of GAI feedback credibility may influence their cognitive engagement, which subsequently contributes to deep learning (Table 1).

**Table 1:** Conceptual distinction among perceived GAI feedback credibility, cognitive engagement, and deep learning.

Dimension	Perceived GAI feedback credibility	Cognitive engagement	Deep learning
Definition	Students’ judgment of the reliability and trustworthiness of GAI feedback information.	Students’ mental effort in processing information during learning.	Students’ achievement of meaningful understanding and knowledge application.
Construct type	Perception	Process	Outcome
Main focus	Evaluation of GAI feedback	Processing of learning information	Quality of learning achieved
Core question	“Is the GAI feedback credible?”	“How do students process the information?”	“What do students achieve from learning?”
Main object	GAI feedback information	Information received during learning	Knowledge and understanding developed through learning
Typical indicators	Reliability, accuracy, trustworthiness	Attention, mental effort, information processing	Understanding, knowledge connection, knowledge application
Does not represent	Actual cognitive processing or learning outcomes	Students’ final learning outcomes	The cognitive effort used during learning

### 2.3 Perceived GAI feedback credibility

Feedback represents an important mechanism that supports learning because it provides students with information about the differences between their current understanding and expected learning goals. Effective feedback can help students identify learning problems, adjust cognitive strategies, and reorganize knowledge structures (Hattie and Timperley, 2007; Shute, 2008). However, feedback itself does not guarantee learning improvement. Previous research suggests that the educational value of feedback depends on whether students actively interpret, evaluate, and use feedback information during learning (Nicol and Macfarlane-Dick, 2006; Carless and Boud, 2018). Therefore, students’ evaluation of feedback information represents an important condition influencing whether feedback can contribute to learning development. From the perspective of source credibility theory, individuals do not accept external information only based on the availability of information. Instead, they evaluate whether information sources possess sufficient reliability, expertise, and trustworthiness before deciding whether to accept and use the information (Hovland and Weiss, 1951). Research on information credibility further indicates that perceived credibility influences individuals’ willingness to consider and apply external information (Flanagin and Metzger, 2000; Appelman and Sundar, 2016). In educational contexts, this suggests that students are more likely to use feedback as a learning resource when they perceive the feedback information as reliable and valuable.

The emergence of GAI has introduced a new information source into learning environments. Unlike traditional teacher feedback, GAI feedback requires students to evaluate information generated by technological systems that may provide useful explanations while also producing inaccurate or incomplete information (Kasneci et al., 2023). Recent studies have shown that students’ acceptance and use of GAI feedback depend on their perceptions of usefulness, reliability, and trustworthiness of GAI systems and their feedback information (Chan and Hu, 2023; Albadarin et al., 2024; Henderson et al., 2025). Moreover, students’ judgments of GAI feedback are influenced by both the characteristics of feedback information and their perceptions of the GAI source (Nazaretsky et al., 2026; Pudasaini, 2026). These findings indicate that perceived GAI feedback credibility may determine whether students regard GAI feedback as meaningful learning information. Deep learning requires students to move beyond information reproduction and develop meaningful understanding, knowledge integration, and knowledge application (Marton and Säljö, 1976). Research on learning approaches further suggests that deep learning occurs when students actively connect new information with existing knowledge structures and construct coherent understanding (Biggs et al., 2001; Entwistle and McCune, 2004). Cognitive learning theories also emphasize that meaningful learning depends on learners’ ability to select, organize, and integrate relevant information into existing cognitive structures (Mayer, 2008). Therefore, the learning value of GAI feedback may depend on whether students perceive the information as credible enough to support such knowledge construction processes.

Recent research on digital learning environments also suggests that learning outcomes depend not only on the availability of technological resources but also on how students interact with and interpret information provided by digital tools (Reinhold et al., 2024; Liu et al., 2026). Studies examining GAI-supported learning further indicate that GAI can influence students’ learning outcomes when students effectively evaluate and use GAI information during learning activities (Han et al., 2025; Chen and Cheung, 2025; Bai and Wang, 2025; Cong-Lem and Fan, 2026; Liang et al., 2026). Therefore, perceived GAI feedback credibility may represent an important psychological factor that determines whether students treat GAI feedback as valuable information for developing deeper understanding. However, perceived GAI feedback credibility does not mean that students blindly accept GAI feedback. Excessive dependence on external information sources may reduce students’ own cognitive effort and lead to cognitive offloading (Risko and Gilbert, 2016). Thus, perceived GAI feedback credibility reflects students’ evaluation of the learning value of GAI feedback rather than passive acceptance. When students perceive GAI feedback as credible, they may be more likely to use the information as a basis for reflection, knowledge integration, and understanding development, thereby supporting deep learning. Accordingly, this study proposes the following hypothesis:

*H1*: Perceived GAI feedback credibility positively influences college students’ deep learning.

Cognitive engagement includes students’ attention, analysis, evaluation, and integration of information during learning (Fredricks et al., 2004; Blumenfeld et al., 2006). Previous research in educational psychology indicates that university students do not allocate the same level of cognitive resources to all information. Instead, they evaluate the value and reliability of information before deciding whether to invest further mental effort (Greene and Miller, 1996; Greene et al., 2004). Therefore, students’ evaluation of learning information may influence their willingness to engage in deeper cognitive processing. In learning environments, cognitive engagement depends not only on whether students receive information but also on whether they actively process and use the information. Previous studies suggest that feedback can support learning when students interpret, evaluate, and apply feedback information during learning activities (Nicol and Macfarlane-Dick, 2006; Carless and Boud, 2018). From a cognitive learning perspective, meaningful learning requires students to select relevant information, organize new knowledge, and connect new information with existing knowledge structures (Mayer, 2008). Therefore, students’ judgments about the value and reliability of feedback information may influence their cognitive investment in processing feedback.

This relationship becomes especially important in GAI-supported learning environments because GAI feedback provides university students with a new type of learning information. Unlike traditional feedback sources, GAI feedback requires university students to evaluate whether the information has sufficient reliability and learning value before they use it. Existing studies indicate that university students’ acceptance and use of GAI feedback depend on their perceptions of usefulness, reliability, and trustworthiness (Chan and Hu, 2023; Albadarin et al., 2024). Henderson et al. (2025) found that university students’ perceptions of usefulness and trustworthiness influence their attitudes toward GAI feedback. Nazaretsky et al. (2026) also showed that university students evaluate feedback differently according to their perceptions of the feedback source. These findings suggest that perceived GAI feedback credibility may influence whether university students are willing to further process GAI feedback.

Based on previous research, perceived GAI feedback credibility may positively influence cognitive engagement because credibility can increase university students’ willingness to invest cognitive effort in processing information. When university students perceive GAI feedback as credible, they may consider the information meaningful and worthy of further examination. This perception may encourage them to compare GAI feedback with their existing knowledge, evaluate the suggestions provided by GAI feedback, and consider how the information can support their learning. These activities represent important features of cognitive engagement because they require active attention, analysis, and knowledge construction (Chi and Wylie, 2014). Research on credibility and information use also supports this relationship. Studies indicate that individuals are more likely to consider and apply information when they perceive the information source as reliable and valuable (Flanagin and Metzger, 2000; Appelman and Sundar, 2016). In educational contexts, students are more likely to treat feedback as a learning resource when they believe that the feedback provides meaningful support for improving their understanding (Carless and Boud, 2018). Therefore, when university students perceive GAI feedback as credible, they may show stronger willingness to examine and interpret the information rather than simply ignore it.

However, perceived GAI feedback credibility does not always lead to active cognitive engagement. Highly credible information may sometimes function as a simple judgment cue, which encourages individuals to accept information without sufficient evaluation (Petty and Cacioppo, 1986). This issue deserves attention in GAI-supported learning environments because excessive dependence on GAI feedback may reduce university students’ own thinking effort and result in cognitive offloading (Risko and Gilbert, 2016). Therefore, perceived GAI feedback credibility should not represent unconditional acceptance of GAI feedback. Instead, it reflects university students’ evaluation of whether GAI feedback provides reliable and meaningful information for learning. This study argues that perceived GAI feedback credibility promotes cognitive engagement when university students use GAI feedback as a learning resource rather than a replacement for their own thinking. Learning with GAI feedback requires university students to understand feedback information, compare it with existing knowledge, judge its relevance, and decide how to apply it. These processes require active cognitive effort rather than simple acceptance. Recent research on GAI-supported learning also suggests that the educational value of GAI depends on how university students evaluate and use GAI information during learning activities (Kasneci et al., 2023; Reinhold et al., 2024; Liang et al., 2026). Studies on GAI learning outcomes further indicate that students’ interaction with and processing of GAI information influence their learning behaviors (Bai and Wang, 2025; Han et al., 2025; Chen and Cheung, 2025; Cong-Lem and Fan, 2026).

In summary, previous studies indicate that students’ perceptions of information credibility influence their willingness to process and use learning information. In GAI-supported learning environments, perceived GAI feedback credibility may encourage university students to invest cognitive resources in examining, evaluating, and integrating GAI feedback. Although excessive trust may cause passive acceptance in some situations, learning feedback requires active processing to support meaningful learning. Therefore, perceived GAI feedback credibility may serve as an important psychological factor influencing university students’ cognitive engagement. Accordingly, this study proposes the following hypothesis:

*H2*: Perceived GAI feedback credibility positively influences college students’ cognitive engagement.

### 2.4 Cognitive engagement

Fredricks et al. (2004) proposed that cognitive engagement represents learners’ willingness to exert effort and employ advanced learning strategies when encountering learning tasks. Similarly, Blumenfeld et al. (2006) argued that cognitively engaged learners are characterized by active meaning construction, strategic thinking, and continuous regulation of their own understanding. Biggs et al. (2001) further suggested that meaningful learning depends on students’ intention to understand concepts, connect ideas, and organize knowledge rather than simply reproduce information. Chi and Wylie (2014) also emphasized that active and constructive cognitive activities are essential for meaningful learning. Therefore, cognitive engagement represents a critical psychological process through which external information is transformed into internal knowledge. Based on this understanding, previous educational research has consistently emphasized that cognitive engagement determines the quality of learning processes rather than merely the amount of learning activities. Early research on learning approaches demonstrated that learners differ fundamentally in how they process information. Marton and Säljö (1976) found that some learners focus primarily on memorizing information and reproducing knowledge, whereas others actively search for meaning, establish connections among concepts, and relate new information to existing knowledge structures. This distinction is consistent with Biggs et al. (2001), who found that deep learning approaches involve active understanding, knowledge organization, and the integration of new information with existing knowledge structures. The latter learning approach, characterized by deeper cognitive involvement, leads to more meaningful and stable understanding. These findings suggest that learning outcomes are not determined only by exposure to information but also by the extent to which learners cognitively process and interpret that information.

Building on this perspective, subsequent studies have further examined how cognitive engagement contributes to effective learning outcomes. Greene et al. (2004) found that students with higher cognitive engagement were more likely to use elaboration strategies, critical thinking, and self-regulated learning behaviors, which subsequently improved their academic achievement. Similarly, Pintrich (2000) highlighted that cognitive engagement is closely associated with self-regulated learning because engaged learners actively monitor their understanding, evaluate their learning progress, and adjust their strategies when necessary. These findings collectively indicate that cognitive engagement functions as a bridge between information exposure and meaningful learning outcomes. Without sufficient cognitive engagement, learners may obtain information but fail to integrate it into their existing knowledge systems.

This mechanism becomes particularly important in GAI-supported learning environments. Unlike traditional learning environments where information is mainly provided through textbooks or instructors, GAI introduces a new form of interactive information source that can generate explanations, suggestions, and feedback according to learners’ questions and needs (Kasneci et al., 2023). However, the availability of GAI-generated information does not automatically guarantee effective learning. Since GAI outputs may contain inaccurate, incomplete, or contextually inappropriate information, learners need to actively evaluate, interpret, and integrate GAI content during interaction (Kasneci et al., 2023). Therefore, cognitive engagement becomes an essential condition determining whether learners can benefit from GAI assistance. When students cognitively engage with GAI feedback information, they are more likely to compare GAI suggestions with their prior knowledge, identify potential limitations, and reconstruct their understanding through reflection and analysis.

Furthermore, cognitive engagement provides the psychological mechanism through which GAI-supported learning can develop into deep learning. Recent studies on GAI-supported learning have suggested that learning outcomes depend on how university students interact with, evaluate, and process GAI information rather than simply receiving information from GAI systems (Han et al., 2025; Chen and Cheung, 2025; Liang et al., 2026). Deep learning emphasizes meaningful understanding, conceptual integration, critical reflection, and the ability to transfer knowledge to new contexts rather than simple information acquisition (Marton and Säljö, 1976). Achieving deep learning requires learners to actively process information by organizing new knowledge, connecting it with existing cognitive structures, and generating personal interpretations. Cognitive engagement directly supports these processes because cognitively engaged learners are more willing to invest mental effort in elaboration, reasoning, and knowledge construction (Blumenfeld et al., 2006). In contrast, when learners interact with GAI systems without cognitive engagement, they may simply accept GAI-generated answers or rely on GAI to complete thinking tasks, which may result in superficial learning rather than deep understanding.

The importance of cognitive engagement is further highlighted by research on cognitive offloading. Risko and Gilbert (2016) argued that external cognitive tools can reduce mental effort by allowing individuals to rely on external information sources instead of internal cognitive processes. Although cognitive offloading can improve efficiency, excessive reliance on external tools may reduce opportunities for active thinking and knowledge development. In the context of GAI-supported learning, this suggests that GAI itself does not determine whether learning becomes deeper or more superficial. Instead, the outcome depends on whether learners actively engage with GAI information and use it as a cognitive support mechanism rather than a substitute for their own thinking. Thus, cognitive engagement represents the key psychological pathway through which GAI-supported interactions are transformed into meaningful learning experiences.

Taken together, previous research suggests that cognitive engagement plays an important role in supporting learning development by encouraging students to actively process learning information. In GAI-supported educational environments, students with higher cognitive engagement are more likely to devote mental effort to examining and processing GAI feedback information rather than passively accepting GAI outputs. Such active processing provides the necessary conditions for learners to make effective use of feedback during learning activities. Therefore, this study proposes that cognitive engagement positively influences college students’ deep learning.

*H3*: Cognitive engagement positively influences college students’ deep learning.

### 2.5 Deep learning

Perceived GAI feedback credibility may influence deep learning not only through a direct pathway but also through a cognitive processing pathway. In learning environments, students’ perceptions of information quality and reliability may shape how they allocate attention and cognitive resources during learning activities. Therefore, cognitive engagement may serve as an important psychological mechanism that explains how perceived GAI feedback credibility is transformed into deeper learning outcomes. Specifically, perceived GAI feedback credibility may first influence students’ willingness to process GAI feedback information, and this cognitive processing may subsequently promote knowledge construction and deep learning.

First, perceived GAI feedback credibility may influence students’ cognitive engagement by shaping their willingness to invest cognitive effort in processing feedback information. Previous research on information credibility suggests that individuals are more likely to consider and use information when they perceive the information source as reliable and valuable (Hovland and Weiss, 1951; Flanagin and Metzger, 2000; Appelman and Sundar, 2016). In educational settings, students are more likely to actively use feedback when they recognize its value and relevance for improving their learning (Nicol and Macfarlane-Dick, 2006; Carless and Boud, 2018). This mechanism may also apply to GAI-supported learning environments. Recent studies have shown that students’ acceptance and use of GAI feedback are influenced by their perceptions of usefulness, reliability, and trustworthiness of GAI information (Chan and Hu, 2023; Albadarin et al., 2024; Henderson et al., 2025). When students perceive GAI feedback as credible, they may be more willing to examine, evaluate, and reflect on the feedback rather than ignore or dismiss it. Such willingness provides the basis for cognitive engagement.

Second, cognitive engagement may serve as the process through which students transform information processing activities into deep learning. Educational psychology research consistently indicates that cognitive engagement involves students’ active effort to analyze information, apply learning strategies, and regulate understanding during learning (Fredricks et al., 2004; Blumenfeld et al., 2006). The ICAP framework further explains that meaningful learning occurs when students actively construct and integrate knowledge rather than simply receive information (Chi and Wylie, 2014). Similarly, studies on learning approaches show that deep learning requires students to connect new information with existing knowledge structures and develop meaningful understanding through active cognitive processing (Marton and Säljö, 1976; Biggs et al., 2001; Entwistle and McCune, 2004). Therefore, cognitive engagement represents a necessary process through which learning information can contribute to deeper understanding.

Third, cognitive engagement may become particularly important in GAI-supported learning because GAI feedback requires students to actively interpret and evaluate information rather than simply accept the provided responses. Research on GAI in education suggests that the educational effects of GAI depend on how students interact with, evaluate, and apply GAI information during learning activities (Kasneci et al., 2023; Albadarin et al., 2024; Yusuf et al., 2024). Recent reviews further indicate that GAI supports learning outcomes when students actively participate in information processing and knowledge construction (Han et al., 2025; Chen and Cheung, 2025; Liang et al., 2026). Meanwhile, research on digital learning environments emphasizes that learning benefits from technology depend on learners’ cognitive activities during interaction with digital tools (Reinhold et al., 2024). Therefore, perceived GAI feedback credibility may promote deep learning by encouraging students to engage cognitively with GAI feedback information. Without such engagement, credible GAI feedback may only result in information acceptance rather than meaningful learning. This perspective also addresses the potential risk of cognitive offloading, where excessive reliance on external information sources reduces individuals’ own cognitive effort (Risko and Gilbert, 2016).

Taken together, perceived GAI feedback credibility may be associated with deep learning through both direct and indirect pathways. Directly, credible GAI feedback can provide meaningful and reliable information that supports students’ understanding and knowledge construction. Indirectly, perceived credibility may stimulate cognitive engagement, through which students actively analyze and integrate GAI feedback information into their learning processes. Therefore, cognitive engagement is proposed as an important mediating mechanism rather than the only pathway linking perceived GAI feedback credibility with deep learning. Based on this reasoning, the following hypothesis is proposed:

*H4*: Cognitive engagement mediates the positive relationship between perceived GAI feedback credibility and deep learning.

The research hypothesis model of this study is presented as follows (Figure 2).

**Figure 2:**
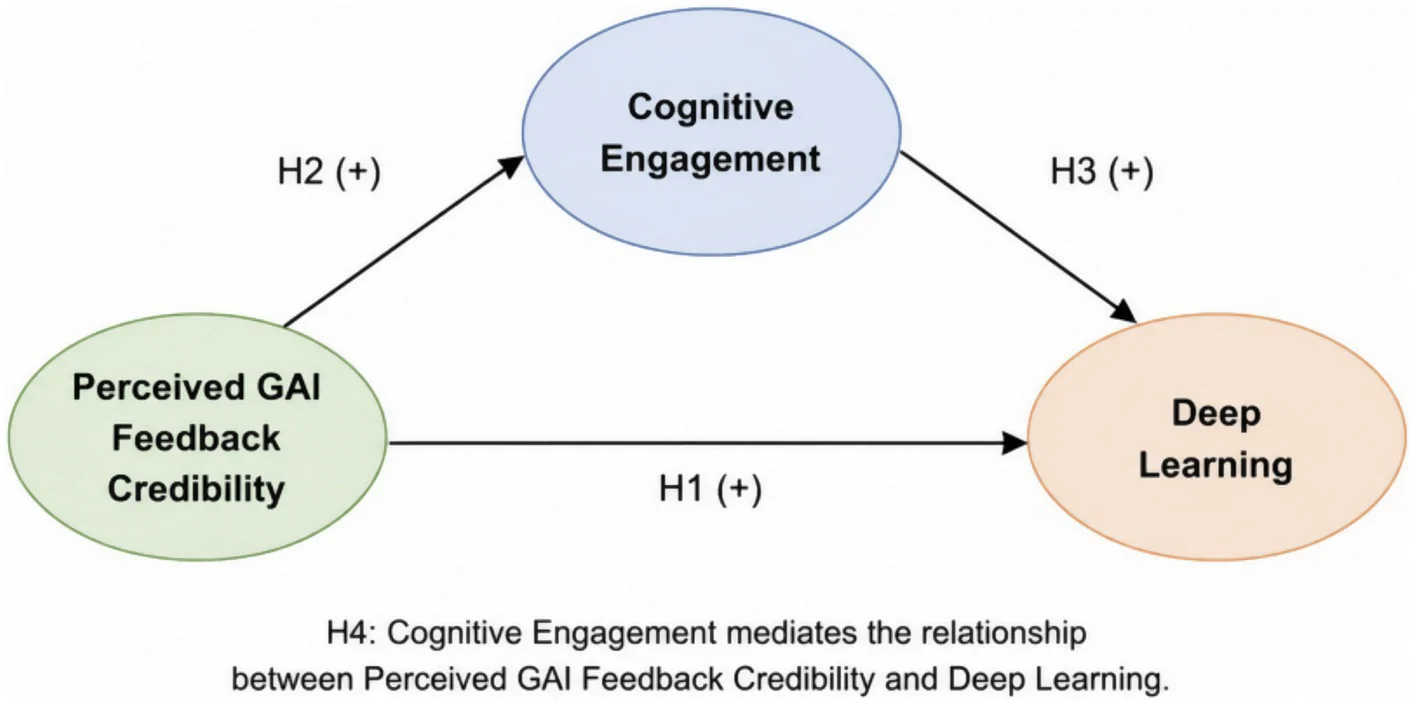
Conceptual model of the relationship between perceived GAI feedback credibility, cognitive engagement, and deep learning.

It is worth noting that the relationship between perceived GAI feedback credibility and deep learning may not be explained only by cognitive engagement. Based on research in educational psychology and learning sciences, learners’ judgments about the credibility of external learning information may influence their learning approaches through different psychological processes. For example, perceiving GAI feedback as credible may increase students’ perceived usefulness of the feedback, making them more willing to use GAI feedback information as a learning resource for understanding and applying knowledge. In addition, credible feedback may strengthen students’ learning confidence and motivation, which may encourage them to adopt deeper learning approaches. Furthermore, students’ recognition of the value of GAI feedback may influence their self-regulated learning processes, such as adjusting learning goals, monitoring learning progress, and selecting appropriate learning strategies. These psychological processes may provide possible explanations for the direct relationship between perceived GAI feedback credibility and deep learning.

However, this study selects cognitive engagement as the mediating mechanism based on the perspectives of Information Processing Theory and feedback research. Unlike simply receiving learning information, the use of GAI feedback requires learners to actively understand, evaluate, and integrate the provided information before it can become personal knowledge. Therefore, whether learners are willing to invest cognitive resources in processing feedback represents an important link between their evaluation of feedback and their learning approaches. When students perceive GAI feedback as credible, they may be more willing to examine the feedback content, evaluate the suggestions provided, and connect the information with their existing knowledge. These activities reflect the core characteristics of cognitive engagement. Therefore, this study does not assume that cognitive engagement is the only psychological pathway through which perceived GAI feedback credibility is associated with deep learning. Instead, cognitive engagement is considered one important theoretically supported mechanism. By examining the mediating role of cognitive engagement, this study aims to explain how learners’ judgments about the credibility of GAI feedback may be related to their active information processing and, subsequently, their deep learning approaches.

## 3 Method

### 3.1 Data source

This study employed a multi-stage stratified sampling method to collect data, aiming to enhance sample representativeness and ensure the reliability of the empirical results. The participants were undergraduate students enrolled in full-time universities in Jinan City, Shandong Province, China. Jinan was selected as the research site for two main reasons. First, as the capital city of Shandong Province, Jinan hosts a diverse range of higher education institutions, including research-oriented universities, comprehensive universities, and application-oriented universities. The diversity of these institutions provides students with different educational backgrounds and levels of exposure to digital technologies, making the city an appropriate context for examining students’ experiences with generative artificial intelligence (GAI). Second, limiting the study to a single geographical area helps reduce the potential influence of regional differences in economic development, educational resources, and digital infrastructure, thereby improving the internal validity of the research model.

In the first stage of sampling, a university sampling framework was established based on university type, educational level, and student population size. Considering that institutional characteristics may influence students’ access to and use of digital technologies, universities were classified into three categories: research-oriented universities, general undergraduate universities, and application-oriented universities. A proportional sampling strategy was then adopted to select universities from different categories. Finally, 10 full-time undergraduate universities in Jinan were selected as the research sites, including 2 research-oriented universities, 6 general undergraduate universities, and 2 application-oriented universities. This distribution was designed based on the overall structure of higher education institutions and the relative proportion of undergraduate students in Jinan. Specifically, general undergraduate universities accounted for 60% of the selected institutions because they represent the largest proportion of undergraduate education providers and therefore reflect the characteristics of the majority of university students. Research-oriented universities accounted for 20% of the sample to capture students with relatively higher exposure to advanced digital technologies, while application-oriented universities also accounted for 20% to represent students from practice-oriented educational environments.

In the second stage of sampling, students within each university were further selected using stratified sampling based on academic year and gender. Each university was assigned approximately 70 questionnaires, resulting in a planned sample size of 700 questionnaires. To ensure balanced representation across learning stages, students from four undergraduate years were included with approximately equal proportions. Specifically, each university distributed 18 questionnaires to first-year students, 18 questionnaires to second-year students, 17 questionnaires to third-year students, and 17 questionnaires to fourth-year students.

The balanced distribution across academic years was designed based on the consideration that students at different stages of university education may have different learning experiences and patterns of GAI use. Compared with junior students, senior students generally possess richer disciplinary knowledge and are more likely to apply GAI tools in complex academic tasks, such as academic writing, professional problem-solving, and research-related activities. Therefore, a sample concentrated in a particular academic year might introduce bias into the findings. Maintaining a relatively equal distribution across academic years allows the study to better capture the general relationship between perceived GAI feedback credibility and students’ cognitive engagement.

Regarding gender distribution, the study controlled the proportion of male and female participants according to the approximate gender composition of undergraduate students in Jinan universities. Rather than relying entirely on convenience sampling, gender quotas were established during the questionnaire distribution process. For example, due to the relatively higher proportion of male students in research-oriented universities, approximately 38 male students and 32 female students were recruited from each research-oriented university. For general undergraduate universities, the distribution was adjusted according to institutional characteristics, with approximately 35 male and 35 female students recruited from each university. Application-oriented universities maintained a relatively balanced gender ratio. Overall, among the 700 planned questionnaires, approximately 360 were allocated to male students (51.4%) and 340 to female students (48.6%), ensuring that the final sample structure was close to the actual undergraduate student population.

In the third stage of data collection, questionnaires were distributed through university instructors, student organizations, and student communication groups. Each participating university appointed one or two student coordinators who were responsible for distributing questionnaires according to the sampling requirements provided by the research team. For example, each university was required to complete approximately 70 valid responses, including 18 first-year students, 18 s-year students, 17 third-year students, and 17 fourth-year students. The coordinators were also instructed to maintain gender balance during distribution to avoid excessive concentration of respondents from a particular gender or academic year. Before completing the questionnaire, all participants were informed of the academic purpose of the study and assured that their responses would be used only for research purposes, reducing potential social desirability bias.

A total of 700 questionnaires were distributed, and 550 responses were collected, resulting in a response rate of 78.57%. After data screening, several procedures were conducted to remove invalid responses. First, questionnaires completed within an unusually short period (e.g., less than 120 s for the entire questionnaire) were excluded. Second, responses showing clear patterns of careless answering, such as selecting the same option for all items, were removed. Third, questionnaires with substantial missing values in key variables were eliminated. Finally, attention-check items were used to identify respondents who did not carefully read the questions. After these screening procedures, 44 invalid responses were removed, leaving 506 valid questionnaires. The effective response rate was therefore 92.00%.

It is worth noting that the questionnaire also included several screening questions regarding participants’ GAI learning experience, including whether they had used GAI tools for learning purposes, the types of GAI tools they had used, the duration and frequency of GAI use, and the primary purposes of using GAI tools in learning activities. These questions were used to identify participants with actual experience of using GAI in learning contexts and to ensure that subsequent evaluations of GAI feedback information were based on real interaction experiences rather than hypothetical situations.

### 3.2 Data analysis method

This study used SPSSAU and AMOS 23.0 for data analysis. During the pilot testing stage, SPSSAU conducted item analysis, reliability analysis, validity analysis, and Harman’s single-factor test, while AMOS 23.0 conducted confirmatory factor analysis (CFA) with the pilot data. During the formal survey stage, SPSSAU conducted reliability analysis, validity analysis, and Harman’s single-factor test, while AMOS 23.0 conducted confirmatory factor analysis (CFA) and structural equation modeling (SEM) analysis with the formal survey data.

Importantly, this study employed Structural Equation Modeling (SEM) to examine the proposed theoretical model and test the hypothesized relationships among the research constructs. SEM was selected because the model involves several latent variables, including Perceived GAI Feedback Credibility, Cognitive Engagement, and Deep Learning. These constructs represent students’ psychological perceptions and learning processes, which cannot be directly measured by a single observable indicator. Instead, they need to be assessed through multiple measurement items. SEM is therefore suitable because it allows researchers to examine both the relationships between latent constructs and their measurement indicators and the relationships among different constructs within one analytical framework.

The use of SEM is particularly appropriate for this study for three reasons. First, the research aims to explore the process through which students’ perceptions of GAI feedback influence their learning behaviors. Specifically, students who perceive GAI feedback information as credible may be more willing to accept and use such feedback, which can further encourage their active involvement in learning tasks. This relationship involves psychological processes that cannot be fully captured by simple correlation analysis. SEM enables the simultaneous examination of multiple relationships, providing a clearer understanding of the proposed theoretical model.

Second, SEM helps address potential measurement errors in psychological research. Variables such as perceived credibility and cognitive engagement are abstract concepts that are usually measured through several questionnaire items. By separating the measurement model from the structural model, SEM allows researchers to first confirm whether the observed items accurately represent the intended constructs and then evaluate the relationships among these constructs. This approach improves the accuracy of the empirical analysis.

Following the two-step procedure of SEM analysis, this study first assessed the measurement model through confirmatory factor analysis (CFA). Reliability and validity tests were conducted to evaluate the quality of the measurement scales, including internal consistency reliability, convergent validity, and discriminant validity. After the measurement model was confirmed, the structural model was examined to test the proposed hypotheses. The significance of path coefficients was evaluated to determine whether Perceived GAI Feedback Credibility had a significant influence on Cognitive Engagement. Moreover, SEM is well suited to the context of GAI-supported learning because students’ responses to GAI feedback information depend not only on the technical performance of the system but also on their perceptions of its credibility and usefulness. Understanding how these perceptions shape students’ learning engagement requires an analytical method that can capture the interaction between technological perceptions and learning behaviors. SEM provides such an approach by linking students’ cognitive evaluations of GAI feedback with their subsequent engagement in learning activities.

### 3.3 Measurement of variables

The measurement instruments used in this study were newly developed based on a review of relevant theoretical and empirical literature. Although previous studies have examined related concepts, such as feedback perception, cognitive engagement, and deep learning, existing instruments were not directly applicable to the context of GAI feedback information in higher education. Therefore, rather than directly adapting existing scales, we developed the items by identifying the core dimensions of each construct from prior studies and tailoring the item wording to the specific context of students’ interactions with GAI feedback. The development process focused on maintaining consistency with the theoretical definitions of the constructs while ensuring that the items could accurately capture students’ perceptions and learning experiences in the GAI-supported learning environment.

Perceived GAI Feedback Credibility was measured as students’ subjective evaluations of whether feedback generated by GAI systems is accurate, relevant, reasonable, dependable, convincing, and capable of increasing their confidence in GAI-generated information. Drawing on previous research on information credibility and trust in intelligent systems, this study focuses on students’ judgments of the credibility-related characteristics of GAI feedback information rather than their intention to use GAI, perceived usefulness of feedback, or learning outcomes. Accordingly, the construct was operationalized through six dimensions of feedback credibility: accuracy, relevance, reasonableness, dependability, convincingness, and confidence (Table 2).

**Table 2:** Measurement items, dimensions, and sources of perceived GAI feedback credibility.

Construct	Dimension	Item Code	Measurement Item	Item development basis
Perceived GAI feedback credibility	Accuracy	GFC1	I believe that the information provided by GAI feedback is accurate.	Developed based on the Flanagin and Metzger (2000)
	Relevance	GFC2	The feedback provided by GAI is consistent with my actual learning situation.	Developed based on the Shute (2008)
	Reasonableness	GFC3	The feedback provided by GAI offers reasonable support for its suggestions.	
	Dependability	GFC4	I consider GAI feedback to be a reliable source of information.	Developed based on the McKnight et al. (2002)
	Convincingness	GFC5	I believe that the explanations provided by GAI feedback are convincing.	Developed based on the McCroskey and Teven (1999)
	Confidence	GFC6	I have confidence in the information provided by GAI feedback.	Developed based on the Flanagin and Metzger (2000)

The first item assessed accuracy, referring to whether students perceived the information provided by GAI feedback as correct. The second item measured relevance, indicating whether the feedback was related to students’ specific learning contexts. The third item captured reasonableness, focusing on whether the feedback was supported by logical reasoning. The fourth item assessed dependability, examining whether students considered GAI feedback a reliable source of information. The fifth item measured convincingness, referring to whether the explanations provided by GAI feedback were persuasive. The sixth item measured confidence, reflecting whether GAI feedback increased students’ confidence in the information provided by the system. All items were measured using a five-point Likert scale ranging from 1 (“strongly disagree”) to 5 (“strongly agree”). These items were developed and refined based on previous studies on information credibility, feedback quality, and trust in artificial intelligence systems to fit the context of GAI-supported learning. By distinguishing credibility from related concepts such as perceived usefulness, behavioral intention, cognitive engagement, and learning outcomes, this measurement captures students’ evaluations of whether GAI feedback information can be considered credible information.

The measurement items for Cognitive Engagement were developed based on previous studies examining students’ mental involvement during learning activities (Table 3). Specifically, this study draws primarily on the ICAP framework proposed by Chi and Wylie (2014), which provides a theoretical basis for understanding differences in students’ levels of cognitive involvement during learning. In addition, items related to students’ mental effort and cognitive strategy use were developed based on the conceptualization of cognitive engagement proposed by Greene and Miller (1996), who examined how students invest cognitive resources during learning tasks. Based on these theoretical foundations, the items were adapted to the context of GAI-supported learning, focusing specifically on students’ mental effort and information processing when interacting with GAI feedback information.

**Table 3:** Measurement items, dimensions, and sources of cognitive engagement.

Construct	Dimension	Item code	Measurement item	Item development basis
Cognitive Engagement	Attention to feedback	CE1	When I receive feedback generated by GAI, I pay attention to the main information in the feedback.	
	Mental effort	CE2	I devote considerable effort to dealing with feedback generated by GAI.	Developed based on the Chi and Wylie (2014)
	Information examination	CE3	I examine the specific content and details of feedback generated by GAI.	
	Feedback consideration	CE4	I evaluate whether GAI feedback information can help me improve my current learning tasks.	
	Feedback review	CE5	Before continuing my learning activities, I carefully review GAI feedback information again.	Developed based on the Greene and Miller (1996)
	Effort persistence	CE6	I continue to use GAI feedback information to support my subsequent learning activities.	

The measurement items for Deep Learning were developed based on previous studies that conceptualize deep learning as a learning outcome (Table 4). Specifically, this study was informed by the deep learning framework proposed by Marton and Säljö (1976), which distinguishes deep learning from surface learning. In addition, the development of the items considered the dimensions of deep learning identified in the Revised Two-Factor Study Process Questionnaire (R-SPQ-2F) developed by Biggs et al. (2001), including seeking meaning, relating concepts, and applying knowledge. Furthermore, the ideas proposed by Entwistle and McCune (2004) regarding deep learning were considered, particularly students’ ability to apply knowledge across different contexts. Based on these theoretical foundations, the measurement items were developed and adapted to the context of GAI-supported learning to assess students’ deep learning outcomes after interacting with GAI feedback information.

**Table 4:** Measurement items, dimensions, and sources of deep learning.

Construct	Dimension	Item code	Measurement item	Item development basis
Deep learning	Meaningful learning	DL1	GAI feedback helps me understand the underlying meaning of the learning content.	Developed based on the Marton and Säljö (1976); Biggs et al. (2001)
	Conceptual connection	DL2	GAI feedback helps me establish connections among different concepts in the learning content.	Developed based on the Biggs et al. (2001)
	Knowledge organization	DL3	GAI feedback helps me develop an overall framework of the learning content.	
	Integrated knowledge use	DL4	GAI feedback helps me combine knowledge from different topics when solving learning tasks.	Developed based on the Entwistle and McCune (2004)
	Knowledge application	DL5	GAI feedback helps me apply what I have learned to solve new problems.	Developed based on the Biggs et al. (2001)
	Knowledge transfer	DL6	I can apply the knowledge gained from GAI feedback to other learning situations.	Developed based on the Marton and Säljö (1976)

### 3.4 Pilot testing and scale validation

Before the formal survey, this study conducted a pilot test with 100 respondents to evaluate the initial quality and applicability of the newly developed measurement scales. Because the scales were developed specifically to capture students’ perceptions and learning experiences related to GAI feedback information, the pilot test was necessary to examine whether the measurement items accurately reflected the theoretical meanings of the constructs and whether any items required further refinement. The pilot test included item analysis (Appendix A), reliability analysis (Appendix B), validity assessment (Appendix C), and exploratory factor analysis (EFA) (Appendix D). The results indicated that the measurement items showed satisfactory psychometric properties, and no substantial issues regarding item clarity, reliability, or construct representation were identified. Based on these findings, this study retained the measurement items and proceeded with the formal survey using a larger sample. The data collected from the formal survey were subsequently used for further validation of the measurement model through reliability analysis, EFA, confirmatory factor analysis (CFA), convergent validity assessment, and discriminant validity assessment before testing the hypothesized relationships.

### 3.5 Descriptive statistics

Based on the results of the pilot test, this study proceeded with the formal survey and collected data from a larger sample. The data used for the descriptive statistics reported in Section 3.5 and all subsequent analyses were obtained from the formal survey. Specifically, the formal survey data were used to further examine the measurement properties of the scales through reliability analysis, EFA, confirmatory factor analysis (CFA), convergent validity assessment, and discriminant validity assessment. After establishing the adequacy of the measurement model, the structural model and hypothesized relationships were examined.

Descriptive statistics were first examined to assess the distributional characteristics of all measurement items. As shown in Table 5, all items were measured using a five-point Likert scale. The median value of all items was 4.000, indicating that students generally tended to agree with the statements regarding their perceptions and learning experiences related to GAI feedback information. The item means ranged from 3.438 to 3.866, with variances ranging from 0.972 to 1.523 and standard errors ranging from 0.044 to 0.055. These results indicate that students generally reported positive evaluations of GAI feedback information, while sufficient variability existed among participants’ responses.

**Table 5:** Descriptive statistics of measurement items.

Name	Variance	Median	S.E	95% CI LL	95% CI UL	IQR	Kurtosis	Skewness	CV
GFC1	1.331	4.000	0.051	3.591	3.792	2.000	−0.593	−0.544	31.245%
GFC2	1.492	4.000	0.054	3.473	3.685	2.000	−0.658	−0.535	34.126%
GFC3	1.343	4.000	0.052	3.589	3.791	2.000	−0.531	−0.555	31.410%
GFC4	1.291	4.000	0.051	3.571	3.769	2.000	−0.504	−0.539	30.958%
GFC5	1.205	4.000	0.049	3.594	3.785	2.000	−0.552	−0.499	29.745%
GFC6	1.362	4.000	0.052	3.556	3.760	2.000	−0.296	−0.674	31.904%
CE1	1.462	4.000	0.054	3.521	3.732	2.000	−0.527	−0.575	33.344%
CE2	1.523	4.000	0.055	3.448	3.663	2.000	−0.642	−0.540	34.707%
CE3	1.463	4.000	0.054	3.547	3.758	2.000	−0.598	−0.562	33.118%
CE4	1.417	4.000	0.053	3.515	3.722	2.000	−0.532	−0.588	32.892%
CE5	1.344	4.000	0.052	3.488	3.690	2.000	−0.405	−0.531	32.297%
CE6	1.358	4.000	0.052	3.438	3.641	1.000	−0.515	−0.488	32.921%
DL1	1.031	4.000	0.045	3.749	3.926	2.000	−0.159	−0.605	26.458%
DL2	0.972	4.000	0.044	3.714	3.886	2.000	−0.180	−0.537	25.942%
DL3	1.174	4.000	0.048	3.771	3.960	2.000	0.060	−0.781	28.029%
DL4	1.093	4.000	0.046	3.743	3.925	2.000	−0.248	−0.593	27.271%
DL5	1.008	4.000	0.045	3.760	3.935	2.000	0.136	−0.740	26.099%
DL6	1.047	4.000	0.045	3.761	3.939	2.000	0.164	−0.743	26.575%

For Perceived GAI Feedback Credibility, the six items (GFC1–GFC6) showed mean scores ranging from 3.556 to 3.692. Among these items, GFC1 (The information provided by GAI feedback is accurate) received the highest mean score (M = 3.692), followed by GFC5 (The explanations provided by GAI feedback are convincing) (M = 3.690) and GFC3 (The feedback provided by GAI offers reasonable support for its suggestions) (M = 3.589). GFC2 (The feedback provided by GAI is consistent with my actual learning situation) obtained a relatively lower mean score (M = 3.579). Overall, these findings suggest that students generally evaluated GAI feedback information positively in terms of accuracy, explanatory quality, and reliability. However, they were relatively more cautious about whether GAI feedback could fully correspond to their individual learning situations. The coefficients of variation for the six items ranged from 29.745 to 34.126%, indicating meaningful individual differences in students’ evaluations of GAI feedback credibility.

For Cognitive Engagement, the six items (CE1–CE6) demonstrated mean scores ranging from 3.438 to 3.652. CE3 (I examine the specific content and details of feedback generated by GAI) obtained the highest mean score (M = 3.652), suggesting that students tended to examine the specific information contained in GAI feedback information during learning activities. CE6 (I continue to use GAI feedback information to support my subsequent learning activities) showed the lowest mean score (M = 3.438), indicating that continuously relying on previously generated feedback to support later learning activities was relatively less common. Overall, these results suggest that students actively engaged with GAI feedback information by attending to and processing the information provided, although differences existed in the extent to which students maintained engagement with feedback over time. The coefficients of variation ranged from 32.297 to 34.707%, indicating adequate response variability across the cognitive engagement items.

For Deep Learning, the six items (DL1–DL6) showed the highest mean scores among the three constructs, ranging from 3.714 to 3.866. DL3 (GAI feedback helps me develop an overall framework of the learning content) received the highest mean score (M = 3.866), followed by DL6 (I can apply the knowledge gained from GAI feedback to other learning situations) (M = 3.761) and DL5 (GAI feedback helps me apply what I have learned to solve new problems) (M = 3.760). DL2 (GAI feedback helps me establish connections among different concepts in the learning content) obtained the lowest mean score (M = 3.714), although it remained above the midpoint of the scale. These findings indicate that students generally perceived GAI feedback information as helpful in supporting their understanding, knowledge organization, application, and transfer of learning. The coefficients of variation ranged from 25.942 to 28.029%, which were lower than those of the other two constructs, suggesting relatively greater consistency in students’ perceptions of the learning outcomes supported by GAI feedback.

The distributional characteristics of all measurement items were further examined. The skewness values ranged from −0.781 to −0.488, while the kurtosis values ranged from −0.658 to 0.164. These values indicate that none of the items showed substantial departures from normality, supporting the suitability of the data for subsequent analyses, including confirmatory factor analysis (CFA) and structural equation modeling (SEM). In addition, the interquartile ranges (IQRs) were mainly between 1 and 2, suggesting that participants’ responses showed sufficient dispersion rather than being concentrated around a single response category.

### 3.6 Sample information

The final sample consisted of 506 undergraduate students from 10 full-time universities in Jinan City. Among them, 258 participants were male (51.0%) and 248 were female (49.0%). Regarding academic year, 127 students were first-year students (25.1%), 126 were second-year students (24.9%), 128 were third-year students (25.3%), and 125 were fourth-year students (24.7%). In terms of university type, 102 participants were from research-oriented universities (20.2%), 304 were from general undergraduate universities (60.1%), and 100 were from application-oriented universities (19.7%). Overall, the final sample demonstrated a relatively balanced distribution across university types, academic years, and gender groups, providing an appropriate foundation for subsequent structural equation modeling and hypothesis testing (Table 6).

**Table 6:** Information of participants.

Characteristics	Category	n	%
Gender	Male	258	51
	Female	248	49
Academic year	First-year student	127	25.1
	Second-year student	126	24.9
	Third-year student	128	25.3
	Fourth-year student	125	24.7
University type	Research-oriented university	102	20.2
	General undergraduate university	304	60.1
	Application-oriented university	100	19.7
Total	—	506	100

To ensure that participants had actual experience with GAI-supported learning, this study also collected information about participants’ GAI usage experience through the personal information section of the questionnaire before measuring the main constructs. All 506 participants reported that they had used GAI tools for learning activities, indicating that their evaluations of Perceived GAI Feedback Credibility were based on actual experiences with GAI systems rather than hypothetical situations.

Regarding the types of GAI tools used in learning activities, participants reported using various GAI platforms. Among them, ChatGPT was the most widely used tool, with 352 students (69.6%) reporting its use, followed by DeepSeek (318 students, 62.8%), Doubao (146 students, 28.9%), Gemini (96 students, 19.0%), and Claude (72 students, 14.2%). In addition, 58 students (11.5%) reported using other GAI tools. Since multiple choices were allowed for this question, the total percentage exceeds 100%.

Regarding the duration of GAI use, 86 students (17.0%) had used GAI tools for less than 3 months, 132 students (26.1%) for 3–6 months, 158 students (31.2%) for 6–12 months, and 130 students (25.7%) for more than 1 year. These results indicate that most participants had accumulated a certain level of experience with GAI-supported learning before participating in the survey.

Regarding the frequency of GAI use, 46 students (9.1%) reported using GAI tools less than once a month, 108 students (21.3%) several times a month, 176 students (34.8%) several times a week, 126 students (24.9%) almost every day, and 50 students (9.9%) multiple times per day. The results suggest that GAI tools had become a relatively common learning support resource among university students.

Regarding the main purposes of using GAI tools in learning activities, participants mainly reported using GAI for understanding course concepts (318 students, 62.8%), receiving explanations about learning materials (342 students, 67.6%), obtaining feedback on assignments or answers (356 students, 70.4%), improving written work or reports (284 students, 56.1%), solving learning problems (301 students, 59.5%), and summarizing course materials (236 students, 46.6%). Since multiple responses were allowed, the percentages do not add up to 100%.

Overall, participants demonstrated relatively rich experience with GAI use in university learning contexts, particularly in activities involving feedback provision, explanation of learning materials, and problem solving. Therefore, the measurement of Perceived GAI Feedback Credibility in this study was based on students’ actual experiences of interacting with GAI feedback information rather than evaluations of an imagined situation. Because some questions regarding GAI usage characteristics allowed multiple responses, the corresponding results are not presented in the sample characteristics table. To maintain consistency and clarity, the Sample Information table only reports basic demographic characteristics of participants, including gender, academic year, and university type. Detailed information regarding participants’ GAI usage experience is presented separately in the text.

### 3.7 Reliability and validity testing

To assess the internal consistency of the measurement instruments, Cronbach’s alpha coefficients and corrected item-total correlations (CITC) were examined. As presented in Table 7, all constructs demonstrated satisfactory reliability, with Cronbach’s alpha coefficients exceeding the recommended threshold of 0.70. Specifically, the Perceived GAI Feedback Credibility (GFC) scale, consisting of six items, achieved a Cronbach’s alpha coefficient of 0.893. The CITC values for the GFC items ranged from 0.691 to 0.758, indicating strong correlations between individual items and the overall construct. The Cognitive Engagement (CE) scale also exhibited excellent internal consistency, with a Cronbach’s alpha coefficient of 0.898 and CITC values ranging from 0.698 to 0.763. The Deep Learning (DL) scale demonstrated good reliability, with a Cronbach’s alpha coefficient of 0.840. The CITC values of the six DL items ranged from 0.592 to 0.653, suggesting that all items contributed adequately to the measurement of the construct. Overall, the reliability analysis indicated that all measurement scales possessed acceptable to excellent internal consistency.

**Table 7:** Reliability analysis of the measurement scales.

Construct	Item	Corrected item-total correlation (CITC)	Cronbach’s α if item deleted	Cronbach’s α
Perceived GAI feedback credibility (GFC)	GFC1	0.733	0.871	0.893
	GFC2	0.758	0.867	
	GFC3	0.696	0.877	
	GFC4	0.706	0.876	
	GFC5	0.698	0.877	
	GFC6	0.691	0.878	
Cognitive engagement (CE)	CE1	0.740	0.878	0.898
	CE2	0.763	0.874	
	CE3	0.724	0.88	
	CE4	0.72	0.881	
	CE5	0.698	0.884	
	CE6	0.698	0.884	
Deep learning (DL)	DL1	0.592	0.818	0.840
	DL2	0.605	0.816	
	DL3	0.637	0.809	
	DL4	0.605	0.816	
	DL5	0.653	0.806	
	DL6	0.605	0.816	

An exploratory factor analysis (EFA) was conducted to examine the construct validity of the measurement scales (Table 8). Prior to conducting the factor analysis, the suitability of the data was assessed using the Kaiser–Meyer–Olkin (KMO) measure and Bartlett’s test of sphericity. The results showed that the KMO value was 0.935, indicating excellent sampling adequacy. Bartlett’s test of sphericity was significant, χ^2^(153) = 4457.807, *p* < 0.001, suggesting that the correlation matrix was appropriate for factor analysis. The EFA results revealed a three-factor structure consistent with the theoretical framework of the study. Three factors with eigenvalues greater than 1 were extracted, explaining 62.553% of the total variance. Before rotation, the three factors accounted for 39.002%, 13.507%, and 10.043% of the variance, respectively. After rotation, the variance explained by the three factors was 21.976%, 21.747%, and 18.829%, respectively. All measurement items showed satisfactory factor loadings, ranging from 0.686 to 0.815, with no item exhibiting cross-loading problems. Specifically, the six Perceived GAI Feedback Credibility (GFC) items loaded strongly on Factor 2, with factor loadings ranging from 0.762 to 0.815. The six Cognitive Engagement (CE) items loaded on Factor 1, with loadings ranging from 0.719 to 0.808. The six Deep Learning (DL) items loaded on Factor 3, with loadings ranging from 0.686 to 0.721. All communalities exceeded 0.50 (range = 0.534–0.718), indicating that the extracted factors adequately explained the variance of each item.

**Table 8:** Validity analysis of the measurement scales.

Item	Factor loading			Communality
	Factor 1	Factor 2	Factor 3	
GFC1	0.141	**0.784**	0.201	0.675
GFC2	0.146	**0.815**	0.157	0.710
GFC3	0.155	**0.762**	0.156	0.629
GFC4	0.119	**0.761**	0.224	0.643
GFC5	0.138	**0.764**	0.173	0.632
GFC6	0.149	**0.767**	0.131	0.627
CE1	**0.805**	0.123	0.158	0.688
CE2	**0.808**	0.103	0.233	0.718
CE3	**0.781**	0.164	0.166	0.664
CE4	**0.793**	0.101	0.155	0.664
CE5	**0.769**	0.162	0.132	0.635
CE6	**0.719**	0.226	0.251	0.631
DL1	0.120	0.153	**0.705**	0.534
DL2	0.201	0.168	**0.686**	0.539
DL3	0.220	0.127	**0.721**	0.584
DL4	0.133	0.173	**0.707**	0.548
DL5	0.223	0.190	**0.713**	0.594
DL6	0.129	0.177	**0.705**	0.546
Initial Eigenvalues (Before Rotation)	7.020	2.431	1.808	-
Percentage of variance explained (before rotation)	39.002%	13.507%	10.043%	-
Cumulative percentage of variance explained (before rotation)	39.002%	52.509%	62.553%	-
Rotated eigenvalues (after rotation)	3.956	3.915	3.389	-
Percentage of variance explained (after rotation)	21.976%	21.747%	18.829%	-
Cumulative percentage of variance explained (after rotation)	21.976%	43.724%	62.553%	-
Kaiser–Meyer–Olkin (KMO) measure	0.935			-
Bartlett’s Test of Sphericity	4457.807			-
Degrees of freedom (df)	153			-
*p*-value	0.000			-

In the table, if numbers are highlighted in bold indicates factor loading values with absolute values greater than 0.40.

### 3.8 Common method bias assessment

To assess the potential influence of common method bias (CMB), Harman’s single-factor test was conducted. All measurement items were entered into an unrotated exploratory factor analysis (EFA). The results indicated that three factors with eigenvalues greater than 1 were extracted, and the first factor accounted for 39.002% of the total variance, which was below the recommended threshold of 40%. Therefore, no substantial common method bias was identified, suggesting that CMB was unlikely to significantly affect the results of this study.

## 4 Results

This study employed structural equation modeling (SEM) to analyze the data. The analysis consisted of two main steps. First, confirmatory factor analysis (CFA) was conducted to evaluate the reliability and validity of the measurement model. Second, the structural model was examined using SEM to investigate the hypothesized relationships among the constructs.

### 4.1 Results of confirmatory factor analysis (CFA)

After assessing the reliability and validity of the measurement scales, confirmatory factor analysis (CFA) was conducted to examine whether the observed items appropriately represented the three latent constructs: Perceived GAI Feedback Credibility (GFC), Cognitive Engagement (CE), and Deep Learning (DL). The CFA was performed using maximum likelihood estimation based on the data from 506 undergraduate students (Table 9).

**Table 9:** Confirmatory factor analysis.

Factor	ITEM	UNSTD	Std. error	C.R	*p*	Std. estimate	SMC	AVE	CR
GFC	GFC1	1.000				0.788	0.621	0.583	0.893
	GFC2	1.089	0.056	19.412	***	0.811	0.658		
	GFC3	0.947	0.054	17.471	***	0.743	0.552		
	GFC4	0.946	0.053	17.861	***	0.757	0.573		
	GFC5	0.896	0.051	17.461	***	0.743	0.551		
	GFC6	0.946	0.055	17.302	***	0.737	0.543		
CE	CE1	1.000				0.785	0.617	0.596	0.898
	CE2	1.060	0.054	19.566	***	0.815	0.665		
	CE3	0.983	0.054	18.319	***	0.772	0.596		
	CE4	0.956	0.053	18.048	***	0.763	0.582		
	CE5	0.905	0.052	17.441	***	0.741	0.549		
	CE6	0.923	0.052	17.752	***	0.752	0.566		
DL	DL1	1.000				0.648	0.419	0.467	0.840
	DL2	1.013	0.080	12.631	***	0.676	0.457		
	DL3	1.163	0.089	13.066	***	0.706	0.498		
	DL4	1.065	0.085	12.547	***	0.670	0.449		
	DL5	1.118	0.083	13.434	***	0.732	0.536		
	DL6	1.035	0.083	12.474	***	0.665	0.442		

***indicates statistical significance at *p* < 0.05.

The results indicated that the proposed three-factor measurement model showed an acceptable fit with the observed data. The chi-square value was 141.245 with 132 degrees of freedom, and the chi-square to degrees of freedom ratio (χ^2^/df) was 1.070, which was below the recommended value of 3. The model also achieved satisfactory values for other fit indices. Specifically, the goodness-of-fit index (GFI) was 0.971, the adjusted goodness-of-fit index (AGFI) was 0.962, the comparative fit index (CFI) was 0.998, the incremental fit index (IFI) was 0.998, the normed fit index (NFI) was 0.969, and the Tucker–Lewis index (TLI) was 0.998. In addition, the root mean square error of approximation (RMSEA) was 0.012, and the standardized root mean square residual (SRMR) was 0.028, both meeting commonly accepted criteria. The 90% confidence interval of RMSEA ranged from 0.011 to 0.025, further indicating that the model estimation was within an acceptable range. These results suggest that the measurement model was consistent with the observed data.

The standardized factor loadings of all measurement items were examined to evaluate the relationship between the observed indicators and their corresponding latent constructs. For Perceived GAI Feedback Credibility, the standardized loadings of GFC1–GFC6 ranged from 0.737 to 0.811. Specifically, GFC2 showed the highest loading (β = 0.811), followed by GFC1 (β = 0.788). For Cognitive Engagement, the standardized loadings of CE1–CE6 ranged from 0.741 to 0.815, with CE2 showing the highest loading (β = 0.815). For Deep Learning, the standardized loadings of DL1–DL6 ranged from 0.648 to 0.732, with DL5 showing the highest loading (β = 0.732). All factor loadings were statistically significant (*p* < 0.001), indicating that each item was significantly associated with its intended construct. The CR values of GFC, CE, and DL were 0.893, 0.898, and 0.840, respectively, indicating satisfactory internal consistency. The AVE values were 0.583, 0.596, and 0.467, respectively. Together with the significant factor loadings of all measurement items, these results indicate that the constructs demonstrated acceptable convergent validity.

In addition, the squared multiple correlation (SMC) values indicated that the measurement items explained a meaningful proportion of variance in their corresponding constructs. The SMC values ranged from 0.543 to 0.658 for GFC items, from 0.549 to 0.665 for CE items, and from 0.419 to 0.536 for DL items. These results suggest that the observed indicators provided adequate measurement information for their respective latent variables. Taken together, the CFA results indicate that the three-factor measurement model demonstrated acceptable model fit, reliability, and validity. The measurement model provided a suitable basis for further analysis of the relationships among Perceived GAI Feedback Credibility, Cognitive Engagement, and Deep Learning.

Discriminant validity was assessed using the Fornell–Larcker criterion by comparing the square roots of the average variance extracted (AVE) values with the correlations among the constructs. As shown in Table 10, the square root of AVE for Perceived GAI Feedback Credibility (GFC) was 0.764, which was higher than its correlations with Cognitive Engagement (CE) (r = 0.388) and Deep Learning (DL) (r = 0.455). Similarly, the square root of AVE for CE was 0.772, exceeding its correlation with DL (r = 0.471). The square root of AVE for DL was 0.683, which was also higher than its correlations with the other constructs. These results indicate that the square roots of AVE for all constructs were greater than their corresponding inter-construct correlations, suggesting that GFC, CE, and DL have adequate discriminant validity and represent empirically distinct constructs.

**Table 10:** Discriminant validity.

	GFC	CE	DL
GFC	0.764		
CE	0.388	0.772	
DL	0.455	0.471	0.683

The values on the diagonal represent the square roots of the AVE values.

Discriminant validity was further assessed using the Heterotrait–Monotrait ratio (HTMT). As shown in Table 11, the HTMT values were 0.434 between GFC and CE, 0.527 between GFC and DL, and 0.543 between CE and DL. All HTMT values were below the recommended threshold of 0.85, indicating satisfactory discriminant validity among the three constructs. These results suggest that although Perceived GAI Feedback Credibility, Cognitive Engagement, and Deep Learning were significantly related, they represented empirically distinct constructs.

**Table 11:** HTMT (Heterotrait–Monotrait ratio) results.

	GFC	CE	DL
GFC	–		
CE	0.434	–	
DL	0.527	0.543	–

### 4.2 Results of structural equation modeling (SEM) analysis

After confirming the reliability and validity of the measurement model, structural equation modeling (SEM) was conducted to examine the proposed relationships among perceived GAI feedback credibility, cognitive engagement, and deep learning. The structural model was estimated using maximum likelihood estimation based on data from 506 undergraduate students. The results showed that the structural model demonstrated satisfactory fit with the empirical data. Specifically, χ^2^ = 141.245 with df = 132, resulting in a χ^2^/df ratio of 1.070. Other fit indices also indicated acceptable model fit, including GFI = 0.971, AGFI = 0.962, CFI = 0.998, IFI = 0.998, NFI = 0.969, and TLI = 0.998. The error indices were also within acceptable ranges (RMSEA = 0.012; SRMR = 0.028). These results indicate that the specified structural relationships were statistically consistent with the observed data. However, because the structural model included all possible paths among the three latent variables, the model was saturated, and the fit indices should not be interpreted as evidence supporting the superiority of the proposed causal ordering over alternative models. Therefore, the following analyses focus on examining the statistical associations among the constructs and the proposed indirect effect rather than establishing causal direction (Table 12).

**Table 12:** Structural equation modeling analysis.

			UNSTD	S.E.	C.R.	*P*	STD
CE	←	GFC	0.472	0.057	8.353	***	0.428
DL	←	CE	0.273	0.038	7.255	***	0.394
DL	←	GFC	0.273	0.041	6.620	***	0.356
GFC6	←	GFC	1.000				0.737
GFC5	←	GFC	0.948	0.058	16.265	***	0.743
GFC4	←	GFC	1.000	0.060	16.587	***	0.757
GFC3	←	GFC	1.001	0.062	16.273	***	0.743
GFC2	←	GFC	1.152	0.065	17.811	***	0.811
GFC1	←	GFC	1.057	0.061	17.302	***	0.788
CE1	←	CE	1.000				0.785
CE2	←	CE	1.060	0.054	19.566	***	0.815
CE3	←	CE	0.983	0.054	18.319	***	0.772
CE4	←	CE	0.956	0.053	18.048	***	0.763
CE5	←	CE	0.905	0.052	17.441	***	0.741
CE6	←	CE	0.923	0.052	17.752	***	0.752
DL1	←	DL	1.000				0.648
DL2	←	DL	1.013	0.080	12.631	***	0.676
DL3	←	DL	1.163	0.089	13.066	***	0.706
DL4	←	DL	1.065	0.085	12.547	***	0.670
DL5	←	DL	1.118	0.083	13.434	***	0.732
DL6	←	DL	1.035	0.083	12.474	***	0.665

***indicates statistical significance at *p* < 0.05.

The standardized path coefficients were then examined to evaluate the hypothesized structural relationships (Figure 3). As shown in Table 12, perceived GAI feedback credibility had a significant positive effect on deep learning (β = 0.356, *p* < 0.001). This finding indicates that students’ perceptions regarding the credibility of GAI feedback information directly contribute to their deep learning outcomes. Specifically, when students perceive GAI feedback as accurate, reliable, and useful, they are more likely to accept the feedback as meaningful learning information and incorporate it into their knowledge construction processes. From a cognitive learning perspective, credible feedback can reduce uncertainty regarding learning tasks, enhance students’ confidence in evaluating their own understanding, and encourage deeper processing strategies, such as reflection, elaboration, and knowledge integration. Therefore, the results provide empirical evidence that perceived GAI feedback credibility represents an important antecedent of deep learning in GAI-supported educational contexts. Thus, H1 was supported.

**Figure 3:**
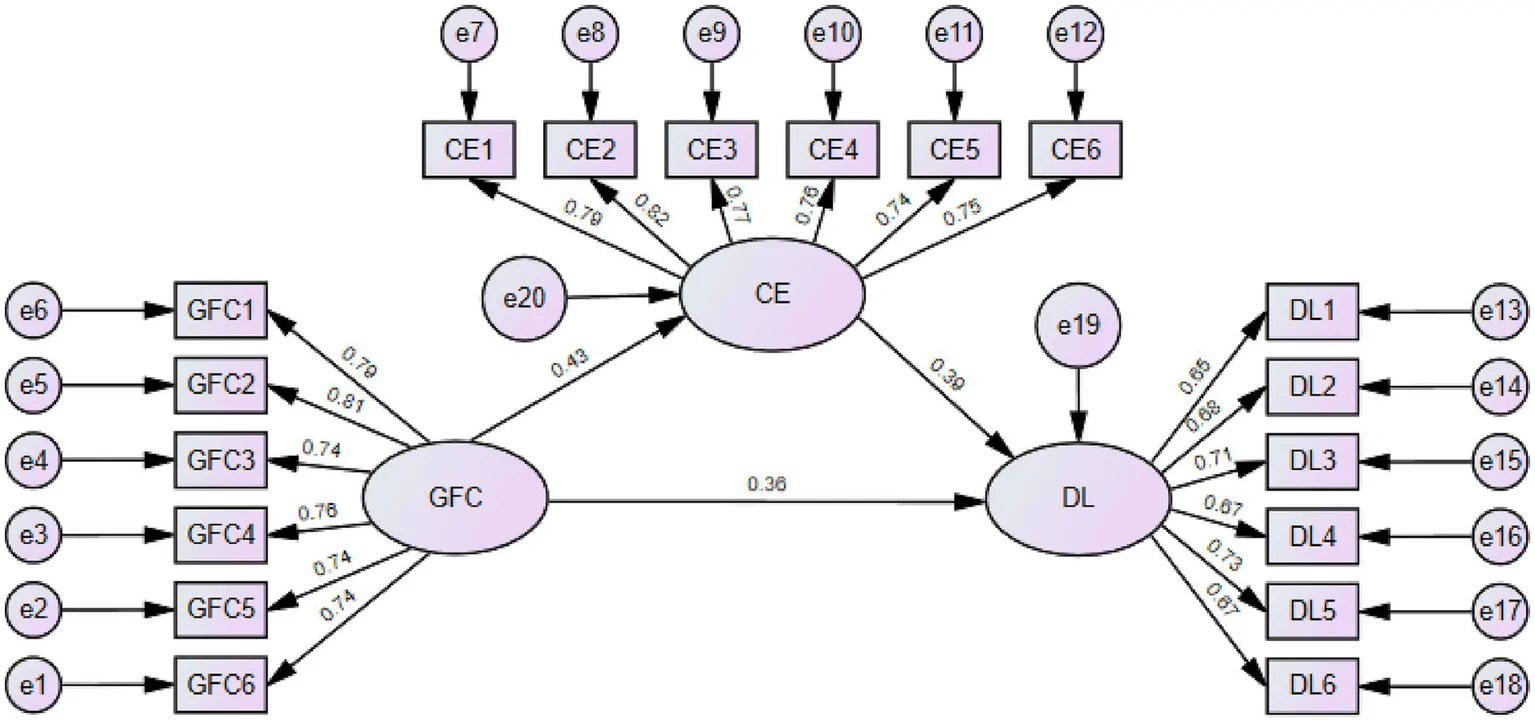
Results of structural equation modeling (SEM) analysis.

In addition, perceived GAI feedback credibility significantly predicted cognitive engagement (β = 0.428, *p* < 0.001), providing support for H2. Among all hypothesized structural paths, this relationship showed the strongest standardized coefficient, suggesting that students’ perceptions of GAI feedback credibility play a particularly important role in stimulating cognitive engagement. This result implies that students are more likely to invest cognitive effort when they trust the information generated by GAI systems. When GAI feedback is perceived as credible, students may be more willing to actively examine feedback content, compare it with their existing knowledge, identify knowledge gaps, and revise their understanding accordingly. Conversely, if students doubt the accuracy or reliability of GAI feedback, they may simply ignore the feedback or engage with it superficially. Therefore, perceived credibility functions as a psychological prerequisite that determines whether students actively process and utilize GAI feedback during learning.

Furthermore, cognitive engagement was found to significantly and positively influence deep learning (β = 0.394, *p* < 0.001), supporting H3. This result demonstrates that students’ active cognitive involvement is a key mechanism underlying deep learning development. Deep learning requires students not only to receive information but also to actively interpret, analyze, evaluate, and connect new knowledge with existing cognitive structures. The significant path from cognitive engagement to deep learning confirms that students who devote greater mental effort to learning activities are more likely to achieve higher levels of conceptual understanding and meaningful knowledge construction. This finding is consistent with cognitive learning theories, which emphasize that learning outcomes depend largely on the depth of learners’ information processing rather than merely on exposure to learning materials.

To further examine the mediating role of Cognitive Engagement in the relationship between Perceived GAI Feedback Credibility and Deep Learning, a bootstrap mediation analysis was conducted within the latent variable structural equation modeling framework using AMOS 31.0. The bootstrap procedure was performed with 2,000 resamples, and bias-corrected percentile confidence intervals were used to assess the statistical significance of the indirect effect. The results showed that the indirect effect of Perceived GAI Feedback Credibility on Deep Learning through Cognitive Engagement was statistically significant (β = 0.168, *p* = 0.001, 95% CI [0.115, 0.232]). Since the 95% bootstrap confidence interval did not include zero, the mediating effect of Cognitive Engagement was supported. This result indicates that Perceived GAI Feedback Credibility was statistically associated with Deep Learning partly through its relationship with students’ Cognitive Engagement. Specifically, Perceived GAI Feedback Credibility was positively associated with Cognitive Engagement (β = 0.428, *p* < 0.001), and Cognitive Engagement was positively associated with Deep Learning (β = 0.394, *p* < 0.001). In addition, the direct effect of Perceived GAI Feedback Credibility on Deep Learning remained significant after Cognitive Engagement was included in the model (β = 0.356, *p* < 0.001). Therefore, Cognitive Engagement played a partial mediating role in the relationship between Perceived GAI Feedback Credibility and Deep Learning.

Furthermore, based on the latent variable SEM results, the total effect of Perceived GAI Feedback Credibility on Deep Learning was calculated as the sum of the direct effect (β = 0.356) and the indirect effect (β = 0.168), resulting in a total effect of β = 0.524. The indirect effect accounted for approximately 32.1% of the total effect. This finding suggests that Cognitive Engagement represents an important pathway through which students’ perceptions of GAI feedback credibility are statistically associated with their deep learning outcomes. This finding suggests that credible GAI feedback influences deep learning through two complementary mechanisms. First, students may directly benefit from high-quality and trustworthy feedback by improving their understanding and correcting misconceptions. Second, credible feedback increases students’ willingness to actively engage in cognitive processing, which further facilitates deeper learning.

The mediation result provides important insights into the psychological mechanism underlying GAI-supported learning. Although generative GAI systems can provide immediate feedback and learning guidance, the educational effectiveness of such feedback does not solely depend on the technological capability of the system. Instead, students’ perceptions of feedback credibility determine whether they actively engage with the provided information. When students trust GAI feedback, they are more likely to allocate cognitive resources toward analyzing and applying the feedback, thereby transforming GAI-generated information into meaningful learning experiences. Thus, the findings extend previous research on technology-enhanced learning by demonstrating that cognitive engagement serves as a critical psychological pathway linking GAI feedback perceptions to learning outcomes.

## 5 Discussion

### 5.1 Theoretical implications

This study makes several theoretical contributions to research on generative artificial intelligence (GAI) in education by shifting attention from what GAI systems can provide to how learners understand and respond to GAI feedback information. Existing studies on GAI-supported education have mainly focused on technological functions, tool adoption, and the possible benefits of GAI applications. However, less attention has been given to the psychological processes through which learners evaluate GAI feedback information and engage with such feedback during learning activities. By introducing Perceived GAI Feedback Credibility as a key psychological construct, this study shows that students’ perceptions of GAI feedback credibility are positively associated with Cognitive Engagement and self-reported Deep Learning. These findings suggest that the educational value of GAI feedback is not simply determined by the ability of GAI systems to generate information, but is also related to whether learners consider such information credible and worthy of cognitive processing. Therefore, this study does not demonstrate that GAI itself directly produces better learning; rather, it explains how students form learning-related associations with GAI feedback information through psychological evaluation and cognitive engagement processes.

First, this study explores the theoretical understanding of GAI-supported learning from an educational psychology perspective by integrating the Elaboration Likelihood Model (ELM), Information Processing Theory, and Constructivist Learning Theory to explain how learners interact with GAI feedback information. Previous research on educational GAI has mainly focused on technological functions, user acceptance, and the potential educational benefits of GAI applications. However, less attention has been given to the psychological processes through which learners evaluate GAI-generated information and transform such information into meaningful learning experiences. By introducing Perceived GAI Feedback Credibility as a key psychological construct, this study demonstrates that learners’ evaluations of GAI feedback credibility are positively associated with Cognitive Engagement and self-reported Deep Learning. These findings suggest that the educational value of GAI feedback does not arise simply from the system’s ability to generate information. Instead, learning-related outcomes depend partly on how learners evaluate, process, and utilize GAI-generated information. Therefore, this study supports the shared assumption of the three theoretical perspectives that external information does not automatically become knowledge; rather, learning requires learners’ active evaluation, cognitive processing, and meaning construction.

Second, this study extends the explanatory application of ELM and Information Processing Theory to GAI-supported learning environments. ELM proposes that individuals’ evaluations of information sources influence their willingness to further process information, while Information Processing Theory emphasizes that meaningful knowledge development requires active cognitive activities, including attention, interpretation, organization, and integration. Consistent with these theoretical perspectives, the findings show that Perceived GAI Feedback Credibility significantly predicts Cognitive Engagement, suggesting that learners’ judgments regarding the credibility of GAI feedback information are related to their subsequent cognitive involvement. Unlike traditional learning environments where information is mainly provided by teachers or human experts, GAI-supported environments introduce GAI systems as new sources of learning information. In this context, learners need to evaluate whether GAI feedback information is reliable and valuable before engaging with it. Thus, this study extends existing cognitive theories by demonstrating how information evaluation and cognitive processing operate in an GAI-mediated learning context.

Third, this study contributes to the application of Constructivist Learning Theory in GAI-supported learning environments. Constructivist perspectives emphasize that learning is not a passive process of receiving external information but an active process in which learners interpret, organize, and integrate new information based on existing knowledge structures. The findings indicate that Cognitive Engagement is positively associated with Deep Learning and partially mediates the relationship between Perceived GAI Feedback Credibility and Deep Learning. This result suggests that GAI feedback information does not directly create deeper learning by itself. Instead, its relationship with deeper learning experiences depends partly on whether learners actively engage with the information and construct meaning from it. Therefore, this study extends constructivist explanations of learning by highlighting the continuing importance of learner cognition in environments where knowledge-related information is increasingly generated by artificial intelligence.

Fourth, this study provides additional insights for research on cognitive engagement and feedback processes in GAI-supported learning environments. It should be noted that cognitive engagement theory and traditional feedback research were not the primary theoretical foundations used to develop the research model; rather, they provide additional perspectives for interpreting the empirical findings. The results show that learners’ responses to GAI feedback involve more than simple acceptance of information. Instead, they involve evaluating feedback credibility, investing cognitive effort, and actively processing GAI-generated information. Furthermore, unlike traditional feedback research that mainly examines feedback provided by teachers or human experts, GAI-supported learning introduces a new feedback source whose reliability requires learner evaluation. Therefore, this study provides further understanding of how feedback processes and learner cognition may operate when feedback is generated by artificial intelligence.

Fifth, this study expands feedback research by examining feedback credibility in the context of GAI-mediated learning. Traditional feedback research has mainly focused on feedback provided by teachers or human experts and has emphasized characteristics such as specificity, timing, and usefulness. However, GAI changes the source of feedback by introducing a new type of feedback provider that can generate immediate explanations, suggestions, and responses. In this context, learners need to evaluate not only the content of feedback but also the credibility of information provided by a GAI system. By identifying Perceived GAI Feedback Credibility as an important factor related to students’ responses to GAI feedback, this study extends feedback research beyond traditional human-to-human interactions and provides a theoretical perspective for understanding feedback processes in GAI-supported learning environments.

Finally, this study provides a new understanding of the learner’s role in GAI-supported learning. Previous discussions of educational technology have often emphasized the functions and capabilities of technological tools, while this study highlights the active role of learners in interpreting and processing GAI feedback information. The findings show that the relationship between GAI feedback and self-reported Deep Learning is not a simple relationship between technological input and learning experience. Instead, it involves learners’ psychological evaluation of feedback and their cognitive engagement with the provided information. This perspective positions GAI-supported learning as a process involving technological information, learner perception, and cognitive processing, rather than merely a process of receiving information from a technological system.

Overall, this study contributes to the theoretical understanding of GAI-supported learning by establishing a learner-centered perspective based on the relationship among Perceived GAI Feedback Credibility, Cognitive Engagement, and Deep Learning. Rather than viewing GAI feedback as an independent factor that automatically improves learning, this study demonstrates that learners’ perceptions and cognitive engagement are important aspects for understanding how GAI feedback information is associated with learning processes. This perspective provides a theoretical basis for further examining the interaction between GAI feedback information and learner cognition in educational contexts.

### 5.2 Practical insights

The emergence of generative artificial intelligence (GAI) is changing the way students interact with learning information. Compared with traditional sources of feedback, GAI can provide immediate explanations, suggestions, and evaluations, creating a new learning situation in which information is no longer provided only by teachers or textbooks but also needs to be actively interpreted and processed by learners. Therefore, in GAI-supported learning environments, the key issue is not simply whether students have access to GAI tools, but how they develop meaningful psychological engagement with GAI feedback information. The practical implication of this study is that the value of GAI feedback as a learning resource depends not only on the information generated by the technology itself but also on how learners understand and process such information. Therefore, educational practices should pay greater attention to learners’ psychological activities during interactions with GAI feedback information rather than focusing only on technological functions.

First, this study suggests that educators should reconsider the abilities students need in the GAI era. Traditional learning environments often emphasize acquiring, memorizing, and applying knowledge, whereas GAI-supported environments require students to interact with explanations and feedback generated by intelligent systems. In this context, learning ability is reflected not only in obtaining information efficiently but also in understanding, analyzing, and using externally generated information appropriately. Educational practices can include reflective learning activities that encourage students to examine the relationship between GAI feedback and their learning goals and to explain how they interpret and use generated information. For example, students may be asked to identify how GAI feedback changes their understanding, which aspects require further consideration, and how the feedback can be incorporated into their own learning process. Such activities may help students treat GAI feedback as a resource for active learning rather than simply as received information.

Second, this study provides practical implications for applying feedback theories in GAI-supported learning contexts. Previous feedback research has mainly focused on feedback provided by human instructors and examined characteristics such as specificity, timing, and usefulness. However, GAI feedback introduces a new psychological issue: how learners understand and respond to information provided by a non-human source. In educational practice, teachers should therefore pay attention not only to whether GAI provides feedback but also to how students participate in the interpretation of such feedback. For instance, teachers can design learning tasks that require students to evaluate the relationship between GAI feedback and their own understanding, explain why they accept or modify certain suggestions, or develop their own ideas based on generated feedback. Such designs may transform feedback from a one-way information delivery process into an active cognitive activity involving learners.

Third, this study highlights the importance of cognitive engagement in GAI-supported learning and provides implications from a psychological process perspective. Learning does not occur simply because information is available; rather, it depends on whether learners actively process and organize information. Therefore, when designing GAI-assisted learning activities, educators should pay attention to whether students are genuinely involved in processing feedback rather than only completing tasks involving GAI. Specifically, learning activities can include opportunities for students to explain, compare, and reflect on GAI feedback information. Such designs may create conditions that encourage students to invest cognitive effort and participate in deeper learning processes.

Fourth, this study provides new insights into the role of teachers in GAI-supported learning environments. The development of GAI does not mean that teachers simply become managers of technology; instead, it highlights the continued importance of teachers as designers of learning experiences and supporters of students’ psychological processes. When GAI becomes part of learning activities, teachers need to pay attention to how students interpret feedback, engage in cognitive processing, and connect external information with their existing knowledge. Therefore, the focus of teachers’ guidance may shift from only delivering knowledge to creating learning environments that encourage explanation, reflection, and active participation.

Overall, the practical significance of this study lies in showing that the value of GAI-supported learning does not depend only on the ability of GAI to generate information, but also on how learners establish psychological connections with generated information. GAI changes the source of learning information, but learning processes still depend on learners’ understanding, processing, and active involvement. Therefore, the application of GAI in higher education should consider learners’ psychological processes and focus on supporting students’ meaningful engagement with GAI feedback information and deeper learning processes.

## 6 Conclusion

Generative artificial intelligence is reshaping the way information is accessed and processed in learning environments. However, a fundamental psychological question remains insufficiently understood: When students encounter GAI feedback information, what determines whether GAI functions as a tool that facilitates cognitive development or becomes a substitute that undermines autonomous thinking? By revealing the psychological pathway of perceived GAI feedback credibility–cognitive engagement–deep learning, the present study provides a new explanation for this question. The findings demonstrate that the educational value of GAI does not originate solely from its information-generating capability; rather, it depends on how learners psychologically evaluate GAI feedback and how such evaluations subsequently influence their cognitive processing. Therefore, this study suggests that future GAI-assisted learning practices should move beyond a focus on “technological effectiveness” toward “psychological effectiveness,” emphasizing the dynamic interplay among trust, engagement, and cognitive activity during human-AI interaction.

First, this study provides important implications for understanding the psychological relationship between students and GAI. Current discussions regarding educational GAI have primarily focused on a technological question: whether GAI can provide accurate, timely, and personalized feedback. However, the present findings indicate that the effectiveness of GAI feedback in promoting learning does not depend solely on the quality of the feedback itself, but also on whether students perceive GAI as a credible source of information. Perceived GAI feedback credibility reflects learners’ psychological evaluation of the value and reliability of GAI-generated information and serves as an important psychological bridge linking technological outputs to learning behaviors. Therefore, in future GAI-enhanced learning environments, educators should not simply encourage students to “trust GAI” or “avoid using GAI”; instead, they should help students develop a more sophisticated trust framework that enables them to evaluate the credibility of GAI feedback based on evidence and contextual relevance. Such calibrated trust can prevent two undesirable outcomes: excessive reliance on GAI suggestions that may lead to cognitive dependency, and insufficient trust that prevents students from benefiting from valuable GAI-based cognitive support.

Second, this study highlights cognitive engagement as a key psychological mechanism connecting GAI feedback experiences with deep learning outcomes. This finding is particularly important because it challenges the technology-deterministic assumption that providing students with more information through GAI will automatically result in improved learning. The results suggest that GAI feedback can facilitate deep learning only when it activates students’ active thinking, analysis, and reflection. From a cognitive psychological perspective, learning is not merely the accumulation of information input; rather, it involves learners’ active selection, processing, organization, and integration of information. Therefore, the primary goal of GAI-assisted learning design should not be to maximize GAI-generated outputs, but to maximize students’ cognitive activities during GAI interaction. For example, GAI systems can encourage deeper information processing by asking students to explain why they accept or reject GAI feedback, compare GAI suggestions with their own perspectives, or anticipate the potential consequences of adopting specific recommendations. In other words, effective GAI-supported learning environments should not enable students to “think less,” but should help them “think better.”

Third, this study provides psychological insights into addressing challenges related to students’ cognitive autonomy in the era of generative GAI. As GAI tools continue to reduce the costs of information searching and knowledge generation, a new educational concern has emerged: students may outsource cognitive tasks to GAI and consequently reduce their own mental effort. However, the present findings suggest that the positive role of GAI does not come from reducing cognitive effort, but from stimulating learners’ cognitive activities through meaningful feedback interaction. Therefore, future GAI-based educational practices should not focus primarily on restricting GAI use; instead, they should cultivate students’ psychological abilities to regulate their interaction with GAI. Specifically, students need to develop metacognitive monitoring abilities that allow them to recognize gaps in their own understanding, evaluate whether GAI feedback aligns with their learning goals, and make informed decisions about when to rely on GAI assistance and when to engage in independent reasoning. Through such psychological regulation, GAI can serve as a tool for expanding human cognitive capabilities rather than replacing human thinking.

Overall, this study advances an important psychological perspective: whether generative GAI promotes learning does not depend on how much information GAI can generate, but on how learners understand, evaluate, and utilize that information. By demonstrating the roles of perceived GAI feedback credibility and cognitive engagement in the development of deep learning, this study provides new insights into the psychological processes underlying human-AI collaborative learning. Future educational practices should therefore focus not only on enabling students to use GAI, but also on helping them establish a productive psychological relationship with GAI, one that promotes autonomous thinking, deep cognitive processing, and sustained intellectual development.

Although this study provides important insights into the psychological mechanisms underlying how perceived GAI feedback credibility promotes deep learning through cognitive engagement, several limitations should be acknowledged.

First, the present study employed a cross-sectional survey design, which limits the ability to draw definitive causal conclusions regarding the relationships among perceived GAI feedback credibility, cognitive engagement, and deep learning. Although the hypothesized model was supported by structural equation modeling and mediation analyses, the findings primarily reflect associations among psychological constructs at a single point in time. Future research could adopt longitudinal designs, experimental approaches, or experience-sampling methods to further examine how students’ trust in GAI feedback develops over time and how changes in GAI-related perceptions influence learning processes.

Second, the sample consisted of undergraduate students from universities in a single Chinese city, which may limit the generalizability of the findings to students from different educational levels, cultural backgrounds, or institutional contexts. Future studies could extend this research by recruiting more diverse samples across different regions and countries, allowing researchers to examine whether cultural values, educational systems, and levels of GAI literacy influence the psychological mechanisms identified in this study.

Third, this study relied primarily on self-reported measures to assess students’ perceptions and learning experiences. Although self-report instruments are widely used in educational psychology research and demonstrated satisfactory reliability and validity in the present study, they may not fully capture students’ actual cognitive behaviors during GAI-assisted learning. Future research could combine self-report data with behavioral indicators, such as GAI interaction logs, learning analytics, task performance measures, or think-aloud protocols, to obtain a more comprehensive understanding of students’ cognitive engagement processes.

Fourth, the measurement scales developed in this study should be further validated in future research. Because GAI-supported learning and GAI feedback information represent relatively emerging educational contexts, existing measurement instruments may not fully capture students’ perceptions and learning processes in this new environment. Therefore, this study operationalized the examined constructs through newly developed scales based on relevant theoretical literature. Before the formal survey, this study conducted a pilot study and performed reliability and validity analyses to examine the preliminary quality of the measures. The formal survey data were further used to assess the measurement model through EFA, CFA, and other validity tests. Nevertheless, these scales have not yet been extensively examined across independent samples, different educational contexts, or diverse GAI application scenarios. Future research should continue to test and refine these measures to further establish their applicability across broader contexts.

Finally, this study did not examine the potential role of GAI usage intensity in the proposed model. Although participants’ GAI usage experience, including usage frequency and duration, was collected through the questionnaire, these characteristics were not incorporated into the current analysis. The present study focused on how students perceive and process GAI feedback information after entering a GAI-supported learning context, rather than on how differences in GAI usage experience shape students’ interactions with GAI systems. However, students with different levels of GAI exposure may develop different levels of familiarity with GAI feedback information, which could influence their perceptions of feedback credibility, cognitive engagement, and learning experiences. Future research could further investigate whether GAI usage intensity functions as a boundary condition by examining its role as a control variable or moderator in the relationship among perceived GAI feedback credibility, cognitive engagement, and deep learning.

## Data Availability

The raw data supporting the conclusions of this article are available from the corresponding author upon reasonable request.
