# Supplementary material for: When college students perceive GAI feedback as credible: the role of perceived GAI feedback credibility in promoting deep learning through cognitive engagement
Source: Front Psychol. 2026 Oct 22;17:1986129. doi: 10.3389/fpsyg.2026.1986129 (PMC13736771; doi:10.3389/fpsyg.2026.1986129)
Supplement: Supplementary file 1 [file Supplementary_file_1.docx]

**APPENDIX**

**Appendix A: Item Analysis of the Pilot Survey Data**

Item analysis was conducted using the pilot survey data to examine whether each measurement item had sufficient discriminative power. Specifically, the scores of all items within each construct were first summed to obtain the total score of the corresponding scale. Based on the total scores, respondents were divided into high-score and low-score groups according to the 27% and 73% percentile criteria. Independent-samples t-tests were then conducted to compare the differences between the two groups for each item. As shown in the tables, all items of the three constructs demonstrated significant differences between the high-score and low-score groups. Specifically, all six items of Perceived GAI Feedback Credibility (GFC1–GFC6), Cognitive Engagement (CE1–CE6), and Deep Learning (DL1–DL6) reached statistical significance (p < 0.01). These results indicate that all items have good discriminative ability and can effectively distinguish respondents with different levels of the measured constructs. Therefore, none of the items were removed, and all items were retained for subsequent reliability and validity analyses (Table A1).

**Table A1.** Item Analysis Results of the Pilot Survey Data

| **Construct** | **Item** | **Low-score group (n=xx) Mean ± SD** | **High-score group (n=xx) Mean ± SD** | **t value** | **p value** |
| --- | --- | --- | --- | --- | --- |
| Perceived GAI Feedback Credibility | GFC1 | 2.46 ± 0.79 | 4.61 ± 0.50 | 12.386 | <0.001 |
|  | GFC2 | 2.25 ± 1.00 | 4.45 ± 0.56 | 10.314 | <0.001 |
|  | GFC3 | 2.04 ± 0.84 | 4.30 ± 0.73 | 11.307 | <0.001 |
|  | GFC4 | 1.89 ± 0.83 | 4.55 ± 0.56 | 14.761 | <0.001 |
|  | GFC5 | 2.32 ± 0.67 | 4.39 ± 0.61 | 12.651 | <0.001 |
|  | GFC6 | 2.36 ± 0.87 | 4.36 ± 0.60 | 10.288 | <0.001 |
| Cognitive Engagement | CE1 | 2.14 ± 0.93 | 4.69 ± 0.54 | 12.563 | <0.001 |
|  | CE2 | 2.29 ± 0.98 | 4.52 ± 0.57 | 10.474 | <0.001 |
|  | CE3 | 2.36 ± 0.73 | 4.38 ± 0.56 | 11.737 | <0.001 |
|  | CE4 | 2.11 ± 0.88 | 4.52 ± 0.63 | 11.941 | <0.001 |
|  | CE5 | 2.43 ± 0.96 | 4.59 ± 0.50 | 10.586 | <0.001 |
|  | CE6 | 2.07 ± 0.98 | 4.38 ± 0.62 | 10.666 | <0.001 |
| Deep Learning | DL1 | 2.79 ± 0.92 | 4.44 ± 0.62 | 8.266 | <0.001 |
|  | DL2 | 2.93 ± 0.81 | 4.53 ± 0.62 | 8.637 | <0.001 |
|  | DL3 | 2.68 ± 1.12 | 4.66 ± 0.48 | 8.640 | <0.001 |
|  | DL4 | 2.75 ± 0.89 | 4.56 ± 0.56 | 9.561 | <0.001 |
|  | DL5 | 2.54 ± 0.79 | 4.44 ± 0.56 | 10.804 | <0.001 |
|  | DL6 | 2.79 ± 0.96 | 4.63 ± 0.49 | 9.168 | <0.001 |

**Appendix B: Reliability Analysis of the Pilot Survey Data**

The reliability analysis was conducted using the pilot survey data. As shown in Appendix B (Table B1), all items demonstrated satisfactory item consistency, with corrected item-total correlations (CITC) exceeding the acceptable level. The Cronbach’s α coefficients for Perceived GAI Feedback Credibility, Cognitive Engagement, and Deep Learning were 0.892, 0.890, and 0.846, respectively. The corresponding standardized Cronbach’s α coefficients were 0.893, 0.890, and 0.847. These results indicate that all three scales demonstrated satisfactory internal consistency reliability, and all items were retained for subsequent analyses.

**Table B1.** Cronbach’s α Reliability Analysis Results of the Pilot Survey Data

| **Construct** | **Item** | **Corrected Item-Total Correlation (CITC)** | **Cronbach’s α if Item Deleted** | **Cronbach’s α** |
| --- | --- | --- | --- | --- |
| Perceived GAI Feedback Credibility | GFC1 | 0.720 | 0.873 | 0.892 |
|  | GFC2 | 0.752 | 0.867 |  |
|  | GFC3 | 0.707 | 0.875 |  |
|  | GFC4 | 0.768 | 0.865 |  |
|  | GFC5 | 0.671 | 0.880 |  |
|  | GFC6 | 0.667 | 0.880 |  |
| Cognitive Engagement | CE1 | 0.732 | 0.867 | 0.890 |
|  | CE2 | 0.725 | 0.869 |  |
|  | CE3 | 0.632 | 0.883 |  |
|  | CE4 | 0.711 | 0.871 |  |
|  | CE5 | 0.705 | 0.872 |  |
|  | CE6 | 0.745 | 0.865 |  |
| Deep Learning | DL1 | 0.589 | 0.828 | 0.846 |
|  | DL2 | 0.572 | 0.831 |  |
|  | DL3 | 0.658 | 0.816 |  |
|  | DL4 | 0.651 | 0.817 |  |
|  | DL5 | 0.672 | 0.813 |  |
|  | DL6 | 0.625 | 0.822 |  |
| *Note: Standardized Cronbach’s α coefficients were 0.893 for Perceived GAI Feedback Credibility, 0.890 for Cognitive Engagement, and 0.847 for Deep Learning.* | | | | |

**Appendix C: Validity Assessment of the Pilot Survey Data**

Validity assessment was conducted using the pilot survey data to examine the construct validity of the newly developed measurement scales (Table C1). Exploratory factor analysis (EFA) was performed to evaluate the underlying factor structure of the measurement items. The results showed that the Kaiser–Meyer–Olkin (KMO) value was 0.825, and Bartlett’s test of sphericity was significant (χ² = 1016.269, df = 153, p < 0.001), indicating that the data were suitable for factor analysis. The EFA results extracted three factors with eigenvalues greater than 1, which corresponded to the three theoretical constructs examined in this study: Perceived GAI Feedback Credibility, Cognitive Engagement, and Deep Learning. After rotation, the three factors explained 63.789% of the total variance. All measurement items showed satisfactory factor loadings, with values exceeding the recommended threshold of 0.40, and the communalities of all items were above 0.40. Specifically, the six items of Perceived GAI Feedback Credibility loaded primarily on Factor 1, the six items of Cognitive Engagement loaded primarily on Factor 2, and the six items of Deep Learning loaded primarily on Factor 3. No substantial cross-loading problems were identified. These results provided preliminary evidence supporting the construct validity of the measurement scales, and all items were retained for the formal survey.

**Table C1.** Exploratory Factor Analysis Results of the Pilot Survey Data

| Item | Factor Loading | | | Communality |
| --- | --- | --- | --- | --- |
|  | Factor 1 | Factor 2 | Factor 3 |  |
| GFC1 | 0.751 | 0.162 | 0.240 | 0.648 |
| GFC2 | 0.805 | 0.136 | 0.112 | 0.679 |
| GFC3 | 0.772 | -0.051 | 0.222 | 0.647 |
| GFC4 | 0.828 | -0.006 | 0.212 | 0.730 |
| GFC5 | 0.798 | 0.096 | 0.023 | 0.647 |
| GFC6 | 0.762 | 0.150 | 0.080 | 0.609 |
| CE1 | 0.093 | 0.794 | 0.180 | 0.672 |
| CE2 | 0.175 | 0.795 | 0.147 | 0.685 |
| CE3 | 0.187 | 0.745 | -0.004 | 0.589 |
| CE4 | -0.054 | 0.801 | 0.162 | 0.671 |
| CE5 | 0.006 | 0.763 | 0.219 | 0.630 |
| CE6 | 0.092 | 0.793 | 0.238 | 0.694 |
| DL1 | 0.014 | 0.213 | 0.698 | 0.532 |
| DL2 | 0.368 | 0.260 | 0.538 | 0.492 |
| DL3 | -0.006 | 0.233 | 0.800 | 0.694 |
| DL4 | 0.200 | 0.093 | 0.745 | 0.604 |
| DL5 | 0.229 | 0.265 | 0.708 | 0.624 |
| DL6 | 0.321 | -0.017 | 0.728 | 0.633 |
| Initial Eigenvalues (Before Rotation) | 6.465 | 3.127 | 1.890 | - |
| Percentage of Variance Explained (Before Rotation) | 35.916% | 17.372% | 10.500% | - |
| Cumulative Percentage of Variance Explained (Before Rotation) | 35.916% | 53.289% | 63.789% | - |
| Rotated Eigenvalues (After Rotation) | 4.126 | 3.997 | 3.359 | - |
| Percentage of Variance Explained (After Rotation) | 22.924% | 22.206% | 18.659% | - |
| Cumulative Percentage of Variance Explained (After Rotation) | 22.924% | 45.130% | 63.789% | - |
| Kaiser–Meyer–Olkin (KMO) Measure | 0.825 | | | - |
| Bartlett’s Test of Sphericity | 1016.269 | | | - |
| Degrees of Freedom (df) | 153 | | | - |
| p-value | < 0.005 | | | - |

Since all variables in this study were measured using self-reported questionnaires from the same respondents, this study conducted Harman’s single-factor test to examine the potential influence of common method bias. All measurement items were entered into an exploratory factor analysis, and an unrotated single-factor solution was applied. The results showed that the first factor explained 35.916% of the total variance, which was below the commonly used threshold of 50%. Therefore, common method bias was unlikely to have substantially influenced the results of this study.

**Appendix D: Exploratory Factor Analysis of the Pilot Survey Data**

Confirmatory factor analysis (CFA) was further conducted using the pilot survey data to examine the preliminary construct validity of the newly developed measurement scales (Table D1). The results showed that all measurement items loaded significantly on their corresponding latent constructs (p < 0.001). The standardized factor loadings ranged from 0.705 to 0.819 for Perceived GAI Feedback Credibility, from 0.662 to 0.811 for Cognitive Engagement, and from 0.630 to 0.760 for Deep Learning. All standardized factor loadings exceeded the commonly accepted threshold of 0.60, indicating that the items demonstrated satisfactory relationships with their intended constructs. These results provided preliminary support for the construct validity of the measurement scales, and all items were retained for the formal survey.

**Table D1.** Confirmatory Factor Analysis Results of the Pilot Survey Data

| **Factor** | **ITEM** | **UNSTD** | **S.E.** | **z** | **p** | **STD** | **SMC** | **AVE** | **CR** |
| --- | --- | --- | --- | --- | --- | --- | --- | --- | --- |
| GFC | GFC1 | 1.000 | - | - | - | 0.781 | 0.610 | 0.584 | 0.894 |
|  | GFC2 | 1.133 | 0.134 | 8.434 | 0.000 | 0.802 | 0.643 |  |  |
|  | GFC3 | 1.128 | 0.143 | 7.882 | 0.000 | 0.757 | 0.573 |  |  |
|  | GFC4 | 1.266 | 0.147 | 8.641 | 0.000 | 0.819 | 0.670 |  |  |
|  | GFC5 | 0.887 | 0.122 | 7.245 | 0.000 | 0.705 | 0.497 |  |  |
|  | GFC6 | 0.933 | 0.127 | 7.360 | 0.000 | 0.714 | 0.510 |  |  |
| CE | CE1 | 1.000 | - | - | - | 0.791 | 0.626 | 0.576 | 0.890 |
|  | CE2 | 0.902 | 0.111 | 8.136 | 0.000 | 0.770 | 0.593 |  |  |
|  | CE3 | 0.755 | 0.111 | 6.796 | 0.000 | 0.662 | 0.438 |  |  |
|  | CE4 | 0.931 | 0.118 | 7.881 | 0.000 | 0.750 | 0.563 |  |  |
|  | CE5 | 0.896 | 0.112 | 8.021 | 0.000 | 0.761 | 0.579 |  |  |
|  | CE6 | 0.954 | 0.110 | 8.657 | 0.000 | 0.811 | 0.657 |  |  |
| DL | DL1 | 1.000 | - | - | - | 0.630 | 0.397 | 0.481 | 0.847 |
|  | DL2 | 0.978 | 0.182 | 5.384 | 0.000 | 0.653 | 0.426 |  |  |
|  | DL3 | 1.250 | 0.219 | 5.716 | 0.000 | 0.707 | 0.499 |  |  |
|  | DL4 | 1.114 | 0.193 | 5.757 | 0.000 | 0.714 | 0.509 |  |  |
|  | DL5 | 1.205 | 0.200 | 6.020 | 0.000 | 0.760 | 0.578 |  |  |
|  | DL6 | 1.118 | 0.199 | 5.611 | 0.000 | 0.689 | 0.475 |  |  |

**Appendix E: Discriminant Validity Assessment of the Pilot Survey Data**

Discriminant validity was further assessed using the Fornell–Larcker criterion based on the pilot survey data. As shown in Appendix E (Table E1), the square roots of the average variance extracted (AVE) for Perceived GAI Feedback Credibility, Cognitive Engagement, and Deep Learning were 0.764, 0.759, and 0.693, respectively. These values were higher than the corresponding correlations between constructs. Specifically, the correlations between Perceived GAI Feedback Credibility and Cognitive Engagement, Perceived GAI Feedback Credibility and Deep Learning, and Cognitive Engagement and Deep Learning were 0.229, 0.427, and 0.431, respectively. Therefore, the results indicate that each construct shared more variance with its own indicators than with other constructs, providing preliminary evidence of discriminant validity.

**Table E1.** Discriminant Validity Assessment Using the Fornell–Larcker Criterion Based on the Pilot Survey Data

|  | GFC | CE | DL |
| --- | --- | --- | --- |
| GFC | 0.764 |  |  |
| CE | 0.229 | 0.759 |  |
| DL | 0.427 | 0.431 | 0.693 |
| *Note: The values on the diagonal (shown in blue) represent the square roots of the average variance extracted (AVE).* | | | |

**Appendix F: English Version of the Questionnaire**

Dear participant,

Imagine that you are working on a course-related learning task. For example, you may be preparing an assignment, writing a course report, solving a problem, or reviewing your answers before submission. During this process, you encounter questions about your work and decide to use a generative artificial intelligence (GAI) tool for assistance. After entering your answers, written content, or learning materials into the GAI system, you receive feedback from the system, such as comments on possible problems, explanations of unclear points, or suggestions for improvement.

Unlike feedback provided by teachers or classmates, GAI feedback information is produced by an artificial intelligence system based on the information you provide. Therefore, when receiving such feedback, students may consider whether the feedback accurately reflects their learning problems, whether it fits their actual learning situation, whether the explanations are reasonable, and whether the information can be trusted as a reference for learning.

This questionnaire aims to understand university students’ experiences and evaluations of GAI feedback information in learning activities. Please recall a specific experience in which you used GAI to obtain feedback on your learning tasks and answer the following questions based on your actual feelings and judgments about the feedback provided by GAI. There are no right or wrong answers. Your responses will be used only for academic research purposes and will be kept confidential.

Part I. Personal Information

1. Gender

☐ Male

☐ Female

2. Academic Year

☐ First-year student

☐ Second-year student

☐ Third-year student

☐ Fourth-year student

3. University Type

☐ Research-oriented university

☐ General undergraduate university

☐ Application-oriented university

4. Have you used GAI tools for learning purposes?

☐ Yes

☐ No

5. Which GAI tools have you used for learning?

(Multiple choices allowed)

☐ ChatGPT

☐ DeepSeek

☐ Gemini

☐ Claude

☐ Doubao

☐ Other: __________

6. How long have you used GAI tools for learning?

☐ Less than 3 months

☐ 3–6 months

☐ 6–12 months

☐ More than 1 year

7. How frequently do you use GAI tools for learning?

☐ Less than once a month

☐ Several times a month

☐ Several times a week

☐ Almost every day

☐ Multiple times per day

8. For what purposes do you mainly use GAI tools in learning?

(Multiple choices allowed)

☐ Understanding course concepts

☐ Receiving explanations about learning materials

☐ Obtaining feedback on assignments or answers

☐ Improving written work or reports

☐ Solving learning problems

☐ Summarizing course materials

☐ Other: __________

Part II. Experiences with GAI feedback information

Please think about your experience of receiving feedback generated by GAI during university learning activities. Please indicate your level of agreement with each statement.

1 = Strongly disagree

2 = Disagree

3 = Neither agree nor disagree

4 = Agree

5 = Strongly agree

1. I believe that the information provided by GAI feedback is accurate.

☐1 ☐2 ☐3 ☐4 ☐5

2. The feedback provided by GAI is consistent with my actual learning situation.

☐1 ☐2 ☐3 ☐4 ☐5

3. The feedback provided by GAI offers reasonable support for its suggestions.

☐1 ☐2 ☐3 ☐4 ☐5

4. I consider GAI feedback to be a reliable source of information.

☐1 ☐2 ☐3 ☐4 ☐5

5. I believe that the explanations provided by GAI feedback are convincing.

☐1 ☐2 ☐3 ☐4 ☐5

6. I have confidence in the information provided by GAI feedback.

☐1 ☐2 ☐3 ☐4 ☐5

7. When I receive feedback generated by GAI, I pay attention to the main information in the feedback.

☐1 ☐2 ☐3 ☐4 ☐5

8. I devote considerable effort to dealing with feedback generated by GAI.

☐1 ☐2 ☐3 ☐4 ☐5

9. I examine the specific content and details of feedback generated by GAI.

☐1 ☐2 ☐3 ☐4 ☐5

10. I evaluate whether GAI feedback information can help me improve my current learning tasks.

☐1 ☐2 ☐3 ☐4 ☐5

11. Before continuing my learning activities, I carefully review GAI feedback information again.

☐1 ☐2 ☐3 ☐4 ☐5

12. I continue to use GAI feedback information to support my subsequent learning activities.

☐1 ☐2 ☐3 ☐4 ☐5

13. GAI feedback helps me understand the underlying meaning of the learning content.

☐1 ☐2 ☐3 ☐4 ☐5

14. GAI feedback helps me establish connections among different concepts in the learning content.

☐1 ☐2 ☐3 ☐4 ☐5

15. GAI feedback helps me develop an overall framework of the learning content.

☐1 ☐2 ☐3 ☐4 ☐5

16. GAI feedback helps me combine knowledge from different topics when solving learning tasks.

☐1 ☐2 ☐3 ☐4 ☐5

17. GAI feedback helps me apply what I have learned to solve new problems.

☐1 ☐2 ☐3 ☐4 ☐5

18. I can apply the knowledge gained from GAI feedback to other learning situations.

☐1 ☐2 ☐3 ☐4 ☐5
